# Cultivating epizoic diatoms provides insights into the evolution and ecology of both epibionts and hosts

**DOI:** 10.1038/s41598-022-19064-0

**Published:** 2022-09-06

**Authors:** Matt P. Ashworth, Roksana Majewska, Thomas A. Frankovich, Michael Sullivan, Sunčica Bosak, Klara Filek, Bart Van de Vijver, Michael Arendt, Jeffrey Schwenter, Ronel Nel, Nathan J. Robinson, Meagan P. Gary, Edward C. Theriot, Nicole I. Stacy, Daryl W. Lam, Justin R. Perrault, Charles A. Manire, Schonna R. Manning

**Affiliations:** 1 Department of Molecular Biosciences, University of Texas, Austin, TX 78712 USA; 2 Unit for Environmental Sciences and Management, North-West University, Potchefstroom, 2520 South Africa; 3 Human Metabolomics, Faculty of Natural and Agricultural Sciences, North-West University, Potchefstroom, 2520 South Africa; 4 Institute of Environment, Florida International University, 11200 SW 8th St., Miami, FL 33037 USA; 5 130 Martinique Drive, Madison, MS 39110 USA; 6 Department of Biology, Faculty of Science, University of Zagreb, Rooseveltov trg 6, 10000 Zagreb, Croatia; 7 Research Department, Meise Botanic Garden, Nieuwelaan 38, 1860 Meise, Belgium; 8 Department of Biology, University of Antwerp, ECOSPHERE, Universiteitsplein 1, 2610 Wilrijk, Belgium; 9 Department of Natural Resources, Marine Resources Division, Charleston, SC USA; 10 Department of Zoology, Institute for Coastal and Marine Research, Nelson Mandela University, Gqeberha, 6031 South Africa; 11 Institut de Ciències del Mar, Spanish National Research Council (CSIC), Barcelona, Spain; 12 Santa Cruz, Institute of Marine Sciences, University of California, Santa Cruz, CA 95060 USA; 13 Department of Integrative Biology, University of Texas, Austin, TX 78712 USA; 14 Department of Comparative, Diagnostic, and Population Medicine, College of Veterinary Medicine, University of Florida, Gainesville, FL 32608 USA; 15 Department of Biological Sciences, University of Alabama, Tuscaloosa, AL 35487 USA; 16 Loggerhead Marinelife Center, Juno Beach, FL 33408 USA

**Keywords:** Speciation, Biodiversity, Microbial ecology

## Abstract

Our understanding of the importance of microbiomes on large aquatic animals—such as whales, sea turtles and manatees—has advanced considerably in recent years. The latest observations indicate that epibiotic diatom communities constitute diverse, polyphyletic, and compositionally stable assemblages that include both putatively obligate epizoic and generalist species. Here, we outline a successful approach to culture putatively obligate epizoic diatoms without their hosts. That some taxa can be cultured independently from their epizoic habitat raises several questions about the nature of the interaction between these animals and their epibionts. This insight allows us to propose further applications and research avenues in this growing area of study. Analyzing the DNA sequences of these cultured strains, we found that several unique diatom taxa have evolved independently to occupy epibiotic habitats. We created a library of reference sequence data for use in metabarcoding surveys of sea turtle and manatee microbiomes that will further facilitate the use of environmental DNA for studying host specificity in epizoic diatoms and the utility of diatoms as indicators of host ecology and health. We encourage the interdisciplinary community working with marine megafauna to consider including diatom sampling and diatom analysis into their routine practices.

## Introduction

Common health indicators currently used to monitor cetaceans, sirenians and sea turtles include mortality rates, demographics, disease prevalence and frequency of stranding events. Since animal-associated microbiota may affect and be affected by their host, both internal and external microbiome composition at any given time could also reflect mid- and longer-term effects of disturbances or stressors experienced by the animal^1^. New health and fitness indices based on compositional changes in the native microbiomes could be a valuable addition to comprehensive health assessments for aquatic vertebrates^2^.

Studies on the external microbiome of large aquatic vertebrates have typically focused on the bacterial and/or viral components. In contrast, epizoic microeukaryotes remain poorly explored despite the observation of diatoms on whales over a century ago^3,4^. Diatoms (Bacillariophyta) are a diverse group of largely photosynthetic microalgae characterized by their uniquely shaped siliceous thecae (frustules) and are commonly found in the plankton and benthos of many different aquatic habitats. Recent studies have expanded the known diversity of epizoic diatoms through increased sampling of hosts to include sea turtles^5–22^, sea snakes^23^ and manatees^24,25^.

Competition for limited resources among diatoms has led to niche partitioning and significant habitat specificity in some taxa. The epizoic diatom communities growing on aquatic vertebrates appear to be formed by a combination of opportunistic surface-attached taxa and putatively obligate epizoic (POE) taxa. While the opportunistic taxa are shared across the benthic habitats of the local environment, the POE taxa thus far have only been observed in the epizoic microbiome^7,21,26,27^. This mixture of opportunistic and POE taxa is an intriguing assemblage, as it is potentially influenced by the host’s biology (e.g. physiology, anatomy and host-specific prokaryotic microbiome) and behavior (e.g. long-distance migrations, diving, basking, and terrestrial nesting which expose epibionts to extremes in temperature, pressure, irradiance, nutrient concentration and desiccation) as well as the environment (e.g. mean temperature, salinity, nutrient load, local biocenoses). Moreover, the unique and highly specific diatom flora composition can be documented long past the death of the diatom cells by the weathering-resistant inorganic frustules. This has resulted in diatoms being utilized extensively for paleoecological reconstructions and bioindication in freshwater environments; for multiple reviews, see^28^. Similar diatom-based health indices may be developed for the marine animals and their habitats.

However, before this can happen, at least two issues must be addressed:

1) We must expand upon our knowledge of the specific molecular, genomic and ecological nature of the interactions between POE diatoms and their host and environment.

2) We need to simplify the identification of epizoic diatoms, which currently requires specialized equipment (such as electron microscopy) and literature that can be highly fragmented and incomplete, particularly in the case of marine diatoms.

Both of these issues could be addressed by metagenomic and metabarcoding techniques, respectively. Currently, however, the dearth of reference data—both in annotated genome and transcriptomes as well as vouchered DNA barcodes for diatoms—would limit the effectiveness of either effort. For example, a metabarcoding attempt on sea turtle epiflora^29^ failed to recover some of the diatom taxa identified in microscopical surveys, including the dominant POE taxon *Labellicula lecohuiana* Majewska, De Stefano & Van de Vijver. The authors acknowledged that this failure was likely due to the lack of any relevant reference sequences for the genus *Labellicula*. Further, the position of *Labellicula* in the molecular phylogeny of diatoms is unknown. This uncertainty significantly hinders any bioinformatic efforts to find sequence data even closely related to *Labellicula* among both the metabarcoding reads and the reference databases. Many other POE taxa have uncertain phylogenetic affinities within the raphid diatoms, including *Tursiocola* Holmes, Nagasawa & Takano, *Epiphalaina* Holmes, Nagasawa & Takano and the “*Tripterion* complex”. This latter assemblage of diatom genera (*Tripterion* Holmes, Nagasawa & Takano, *Chelonicola* Majewska, De Stefano & Van de Vijver, *Poulinea* Majewska, De Stefano & Van de Vijver and *Medlinella* Frankovich, Ashworth & M.J.Sullivan) is of particular taxonomic interest as they represent a radiation of exclusively epizoic diatom taxa. Their current taxonomy is not universally accepted^15^, and distinguishing the genera can be difficult without the use of electron microscopy due to a similar overall frustule morphology (heteropolar, stalked and septate or pseudoseptate) and relatively small size (< 20 um).

To address the aforementioned issues, we have cultured and sequenced DNA data from POE diatom taxa. These were isolated from sea turtles and manatees from the wild, rehabilitation and rescue centers as well as aquaria from the United States of America, The Bahamas, Croatia, Italy and South Africa. While DNA sequence data from vouchered specimens alone would be useful for molecular identification, the ability to maintain these diatoms away from their hosts facilitates the formulation of hypotheses and laboratory experiments to test the molecular nature of the relationship between the diatom and host.

## Results

### Culture success

**Figure 1:**
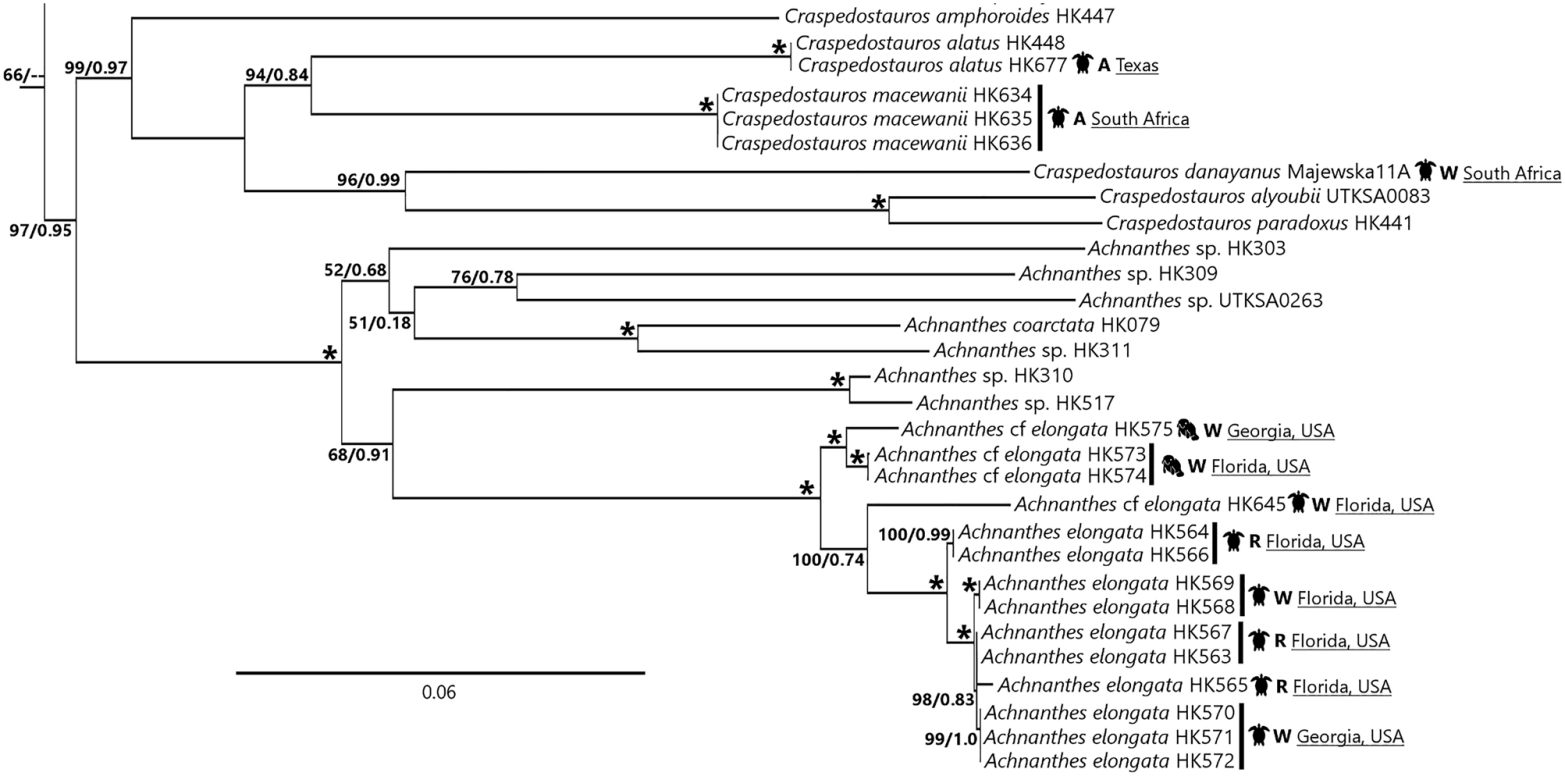
Maximum likelihood phylogenetic tree derived from a concatenated 3-gene DNA sequence dataset, representing the *Achnanthes*, *Craspedostauros* and *Staurotropis* clades (complete tree shown in Fig. S1). Support values (ML bootstrap support/BI posterior probability) shown above nodes; “*” = nodes with 100%/1.0 values. Taxon name followed by DNA extraction voucher number or strain ID. Taxa isolated from epizoic habitats followed by a diagrammatic representation of the host from which the strain was isolated, and metadata on the location and setting in which the host was sampled (A = aquarium, R = rehabilitation facility, W = wild).

**Figure 2:**
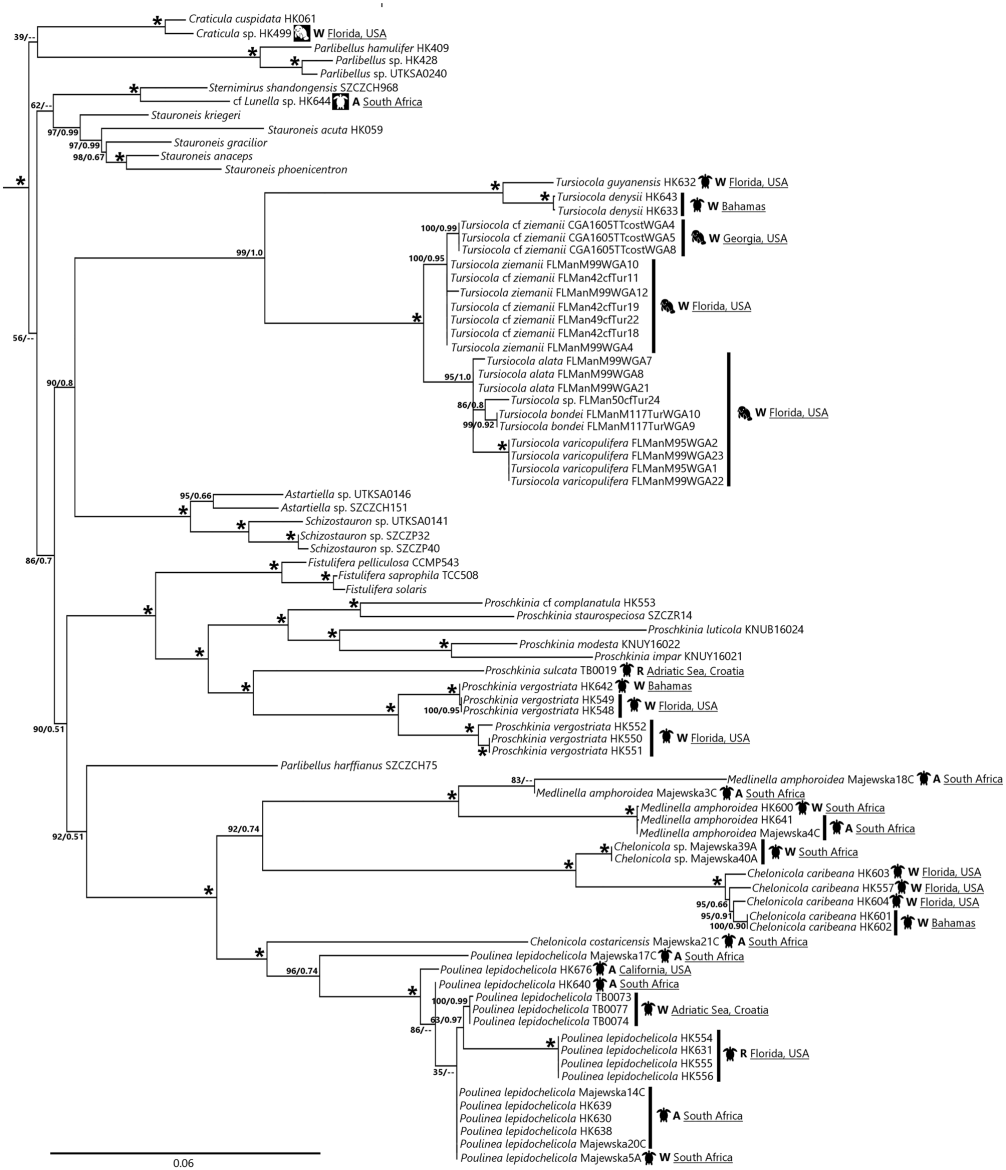
Maximum likelihood phylogenetic tree derived from a concatenated 3-gene DNA sequence dataset, representing the clade containing the *Tripterion* complex, *Tursiocola* and *Proschkinia* clades (complete tree shown in Fig. S1). Support values (ML bootstrap support/BI posterior probability) shown above nodes; “*” = nodes with 100%/1.0 values. Taxon name followed by DNA extraction voucher number or strain ID. Taxa isolated from epizoic habitats followed by a diagrammatic representation of the host from which the strain was isolated, and metadata on the location and setting in which the host was sampled (A = aquarium, R = rehabilitation facility, W = wild). Black host icon = POE taxon; white host icon = unclear habitat preference.

We successfully cultured > 600 strains, both POE and opportunistic diatoms on the epizoic habitat. This manuscript focuses on 76 of these sequenced strains (Table 1) and the sequences from the single-cell DNA extractions of the non-photosynthetic *Tursiocola* spp. (Figs. 1, 2). Sequence data from 21 additional diatoms are included (Figs. S1, S2). While these additional sequenced diatom taxa were isolated from epizoic collections, they are known opportunistic taxa, occur in non-epizoic habitats, or their habitat preferences are unclear.

**Table 1:** POE diatoms cultured in this study, sorted by host species.

Host species	Location	Host status	POE Diatoms cultured (# of strains) [total cultures]
*Chelonia mydas (Green Sea Turtle)*	Bahamas	Wild animal: “turtle1”	Cca (2), Td (2), Pv (1) ^9^
*Chelonia mydas (Green Sea Turtle)*	Durban, South Africa	Aquarium resident: “Calypso”	Cco (1), Cm (3), Pl (2) ^14^
*Chelonia mydas (Green Sea Turtle)*	Durban, South Africa	Aquarium resident: “Wasabi”	Ma (1), Pl (4) ^12^
*Chelonia mydas (Green Sea Turtle)*	Florida, USA	Wild animal: “FL noname”	Ae (2), Tg (1) ^6^
*Chelonia mydas (Green Sea Turtle)*	Florida, USA	Rehabilitation animal: “Fleming”	Ae (5), Pl (3), Pv (3) ^22^
*Eretmochelys imbricata (Hawksbill Sea Turtle)*	Texas, USA	Aquarium resident: “Einstein”	Ca (2) ^4^
*Eretmochelys imbricata (Hawksbill Sea Turtle)*	Durban, South Africa	Aquarium resident: “Tripod”	Ma (3) ^11^
*Lepidochelys kempii (Kemp's Ridley Sea Turtle)*	Georgia, USA	Wild animal: “Z6”	Ae (3) ^12^
*Dermochelys coriacea (Leatherback Sea Turtle)*	Kosi Bay, South Africa	Wild animal: “ZA0019A/ZA1824E”	Cd (1) ^7^
*Caretta caretta (Loggerhead Sea Turtle)*	Durban, South Africa	Aquarium resident: “Shiv”	Pl (3) ^6^
*Caretta caretta (Loggerhead Sea Turtle)*	Kosi Bay, South Africa	Wild animal: “ZA00940/ZA10860”	Ma (1) ^4^
*Caretta caretta (Loggerhead Sea Turtle)*	Kosi Bay, South Africa	Wild animal: “ZA1595E/ZA1826E”	Pl (1) ^10^
*Caretta caretta (Loggerhead Sea Turtle)*	Kosi Bay, South Africa	Wild animal	Csp (2) ^10^
*Caretta caretta (Loggerhead Sea Turtle)*	Florida, USA	Wild animal: “A2”	Ae (7) ^8^
*Caretta caretta (Loggerhead Sea Turtle)*	Florida, USA	Wild animal: “CC032217a”	Cca (2), Pv (2) ^19^
*Caretta caretta (Loggerhead Sea Turtle)*	Florida, USA	Wild animal: “FL Christine”	Cca (2) ^6^
*Caretta caretta (Loggerhead Sea Turtle)*	Brijuni Islands, Croatia	Aquarium resident: “Lunga”	Ps (1) ^3^
*Caretta caretta (Loggerhead Sea Turtle)*	Bisceglie, Italy	Rehabilitation animal: “Iracus”	Pl (3) ^38^
*Lepidochelys olivacea (Olive Ridley Sea Turtle)*	Long Beach, California	Aquarium resident: “LoMain”	Pl (1) ^14^
*Lepidochelys olivacea (Olive Ridley Sea Turtle)*	Florida, USA	Rehabilitation animal: “Harry”	Ae (2), Pl (3) ^10^
*Trichechus manatus latirostris (West Indian Manatee)*	Florida, USA	Wild animal: “FLMan40”	Ae (2) ^11^
*Trichechus manatus latirostris (West Indian Manatee)*	Georgia, USA	Wild animal: “CGA1605”	Ae (1) ^23^

POE diatoms are abbreviated and followed by the number of strains cultured from the indicated host: Ae = *Achnanthes elongata*, Ca = *Craspedostauros alatus*, Cd = *Craspedostauros danayanus*, Cm = *Craspedostauros macewanii*, Cca = *Chelonicola caribeana*, Cco = *Chelonicola costaricensis*, Csp = *Chelonicola sp*., Ma = *Medlinella amphoroidea*, Pl = *Poulinea lepidochelicola*, Ps = *Proschkinia sulcata*, Pv = *Proschkinia vergostriata*, Td = *Tursiocola denysii*, Tg = *Tursiocola guyanensis.*

### Target POE taxa

**Figure 3:**
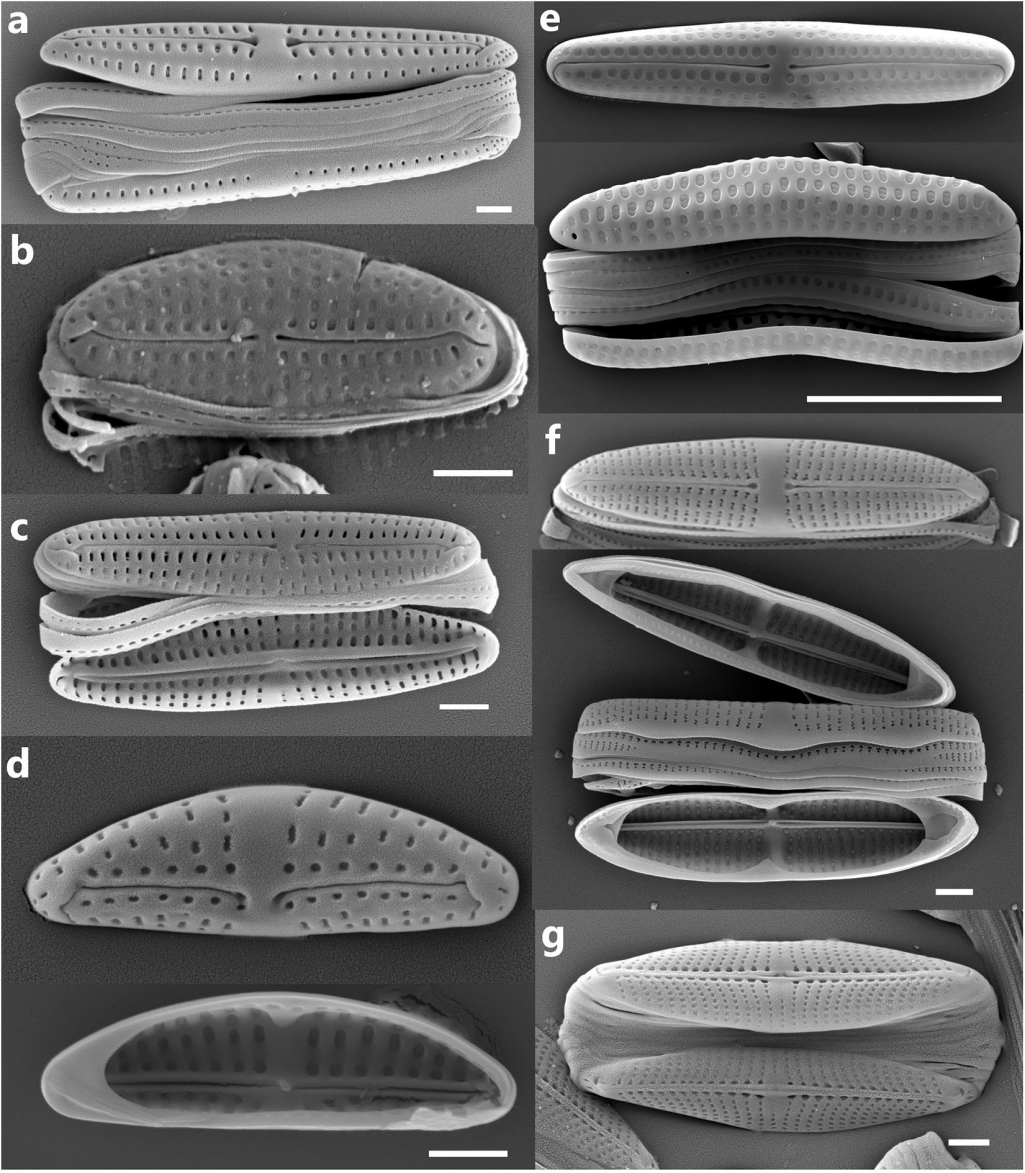
Scanning electron micrographs of some of the POE diatom taxa successfully cultured and sampled for DNA. a = *Poulinea lepidochelicola* HK630, complete frustule. b = *Chelonicola* cf *costaricensis* Majewska 21C, valve exterior. c = *Chelonicola* sp. Majewska 40A, complete frustule. d = *Medlinella amphoroidea* HK600 (valve exterior above, interior below). e = *Achnanthes elongata* HK563 (valve exterior above, complete frustule below). f = *Tursiocola denysii* HK633 (valve exterior above, complete frustule below). g = *Proschkinia vergostriata* HK552, complete frustule. All scale bars = 1 µm.

POE taxa were identified based on the available literature and included diatom species that have only ever been observed in association with the epizoic habit being found on multiple animal specimens^6,8,10,11,14–16,24,25,30^. Among these were epizoic taxa typically reaching high relative abundances (> 25%)—*Achnanthes elongata* Majewska & Van de Vijver, *Chelonicola costaricensis* Majewska, De Stefano & Van de Vijver, *C. caribeana* Riaux-Gobin, Witkowski, Ector & Chevallier, *Craspedostauros danayanus* Majewska & Ashworth, *Medlinella amphoroidea* Frankovich, Ashworth & M.J.Sullivan, *Poulinea lepidochelicola* Majewska, De Stefano & Van de Vijver, *Tursiocola* spp., as well as species often present on animals but never exceeding 10% of the diatom relative abundance—*Craspedostauros alatus* Majewska & Ashworth, *C. macewanii* Majewska & Ashworth, *Proschkinia sulcata* Majewska, Van de Vijver & Bosak and *P. vergostriata* Frankovich, Ashworth & M.J Sullivan. SEM images of some of these taxa sampled for DNA can be found in Fig. 3. This list of POE taxa is not exhaustive as the full diversity of POE diatoms remains to be documented. Moreover, it does not include several probable POE species (e.g. *Achnanthes squaliformis* Majewska & Van de Vijver, *Navicula dermochelycola* Riaux-Gobin, Witkowski, Kociolek & Chevallier), which have not yet been isolated and cultured.

### Molecular phylogenetic results

The currently recognized POE strains were predominantly located in two clades in the molecular phylogeny—*Achnanthes* sensu stricto + *Craspedostauros* (Fig. 1) and the clade containing the *Tripterion* complex, *Tursiocola* and *Proschkinia* (Fig. 2). With regards to *Achnanthes*, most of the sampled diversity comes from three species of sea turtles (green, Kemp’s ridley and loggerhead) and West Indian manatees sampled in the southeastern US. These strains formed a well-supported clade (ML bootstrap support [bs] = 100%, BI posterior probability [pp] = 1.0) sister to the rest of the sequenced *Achnanthes* spp. The POE *Achnanthes* clade also sorted by host, with strains collected from manatee (100%/1.0 bs/pp) and sea turtle (100%/0.74 bs/pp) hosts in their own clades. The POE *Craspedostauros* taxa showed a different pattern to the rest of the POE diatoms. Their clade included both POE and non-POE species, with POE taxon *C. danayanus* sister to *C. alyoubii* and *C. paradoxus* (96%/0.99 bs/pp) rather than to the POE *C. macewanii* and *C. alatus*.

The “*Tripterion* complex + ” clade (strains illustrated in Fig. 3a–d) was resolved with strong support (100%/1.0 bs/pp). While we were able to sample taxa from the *Chelonicola*, *Poulinea* and *Medlinella* genera in this complex, we were unable to observe any taxa within *Tripterion* sensu stricto in our collections. The “*Tripterion* complex + ” clade also contained the POE genus *Tursiocola* and *Proschkinia* Karayeva, which has both POE and non-POE species, as well as the non-epizoic genera *Stauroneis* Ehrenberg, *Craticula* Grunow, *Parlibellus* E.J.Cox, *Fistulifera* Lange-Bertalot and some monoraphid genera such as *Schizostauron* Grunow and *Astartiella* Witkowski, Lange-Bertalot & Metzeltin. The molecular data suggested no common origin for the POE clades; *Tursiocola* and the *Tripterion* complex are sister to non-POE taxa rather than each other, and the POE *Proschkinia* (*P. vergostriata* and *P. sulcata*) formed a clade sister (100%/1.0 bs/pp) to the rest of the *Proschkinia* spp.

Within *Tursiocola*, both nutritional types appear monophyletic, with the non-photosynthetic manatee-associated taxa (*T. alata*, *T. bondei*, *T. varicopulifera* and *T. ziemanii*) and the photosynthetic sea turtle-associated taxa (*T. denysii* and *T. guyanensis*) in their own clades (100%/1.0 bs/pp for both clades). It should be noted, however, that there were only two photosynthetic *Tursiocola* taxa sampled. Tree topology in the *Tripterion* complex remained the same regardless of analysis, with *Chelonicola costaricensis* “Majewska21C” + *Poulinea lepidochelicola* (100%/1.0 bs/pp) sister to *Medlinella amphoroidea* + *Chelonicola* sp. “Majewska39A/40A” + *C. caribeana* (92%/0.74 bs/pp).

Only two clades in the *Tripterion* complex had any geographic variation: the *Poulinea* clade and *Chelonicola caribeana* clade. For *Poulinea*, strains collected in South Africa were not monophyletic, with “Majewska 17C” sister to the rest of the clade, which included strains isolated from the Adriatic, Florida, California and South Africa. It should be noted that the Florida clade represented strains collected from a single location—a rehabilitation facility—while the South African strains were isolated from collections of both wild and captive host animals. The *C. caribeana* clade, on the other hand, contained strains isolated exclusively from wild host animals in South Africa, Florida and the Bahamas, with the South African strains (“Majewska39A/40A”) sister to the rest.

## Discussion

Based on our molecular phylogeny, it appears that the epizoic habit has evolved several times and in several different raphid diatom morphotypes: elongate biraphid (*Tursiocola* and *Proschkinia* , Fig. 3f,g, respectively) and monoraphid frustules (*Achnanthes*, Fig. 3e), asymmetric, clavate biraphid frustules (*Tripterion* complex, Fig. 3a) and thin oval monoraphid frustules (*Bennettella, Epipellis*^31^). These independent gains of the epizoic habit could be driven by the host biology and evolution. The various epizoic diatom lineages, if eventually resolved to be closely linked to a specific type of host animal, might have diverged from non-epizoic taxa under different ecological and evolutionary constraints and at different times corresponding to the emergence of various groups of marine megafauna.

Among others, the eco-physiological constraints shaping epizoic diatom speciation through adaptive radiation would include the nature and character of the animal substrate. Variations of the dermal layer of sirenians and sea turtles including the ultrastructure, topology, physiology (e.g. shedding patterns), and biochemistry (e.g. enzymatic activity) would require different attachment and colonization (and re-colonization) strategies, thus encouraging the development of specific adaptations. Such a specific adaptation is evidenced by *Melanothamnus maniticola* Woodworth, Frankovich & Freshwater, an epizoic red alga on manatees that has unique skin penetrating rhizoids that anchor the thallus to the deeper epidermis and permit the alga to persist as the host surface skin cells are shed^32^. In marine reptiles, the carapace scutes are often shed periodically, while the skin scales are either shed continuously (sea turtles) or the epidermis is renewed completely in a process called ecdysis (sea snakes^33^). These patterns differ from those observed in marine mammals in which skin shedding may be regulated by external factors such as temperature^34^. Similarly, animals with different diving regimes may host diatoms with different physiological and metabolic adaptations as various stages of photosynthesis will be differently affected by changes in hydrostatic pressure related to the depth, duration, and frequency of dives^35^.

Moreover, the diversification dynamics in POE diatoms may be linked to the host animal behavior and lifestyle. The niche heterogeneity, biodiversity, productivity, and nutrient concentrations typical of shallow-water habitats occupied by sirenians and some sea turtles may increase colonization rates by new species and favor benthic diatom immigration to the epizoic community, thus spurring the observed diversity of diatom forms associated with manatees^24,25^ or sea turtles using neritic foraging habitats (e.g. loggerheads;^21^). The opposite phenomenon could explain low epizoic diatom diversity on leatherback sea turtles^5,30^, and pelagic sea snakes^23^ that spend significant time feeding in the pelagic zone rather than on benthic organisms^36^. This follows the general pattern of low macro-epibiotic diversity on leatherbacks^37^. Epizoic diatom diversity might also be driven by intrinsic biotic factors, such as gregariousness and range of the host species as both factors may affect the new species encounter and colonization rates. However, in these systems in which epizoic diatom species richness is driven mainly by speciation rates as opposed to benthic species immigration, the total epizoic diatom diversity may remain low. The higher number of diatom taxa observed on neritic megafauna species as compared to open-water animals seem to support this hypothesis^20^.

Currently, taxon sampling is still scattered, and while strains were isolated from multiple geographic localities, much of the strain diversity in species-level clades come from a single collection. The Florida *Poulinea lepidochelicola* clade, for example, represents strains isolated exclusively from the Turtle Hospital rehabilitation facility in Marathon, Florida. Among the South African *P. lepidochelicola* strains, six strains (Majewksa 14C, Majewska 20C, HK630, HK638, HK639 and HK640) came from collections from three turtles at the uShaka Sea World facility in Durban, and likely represent one population. However, a morphological difference does exist between the sequenced *Medlinella amphoroidea* strains from South Africa and the type population of Florida Bay. The valve areolae of the former appear to be occluded by hymenes (Fig. 3d) as opposed to the volae of the type population^14^. Whether this corresponds to a genetic, and perhaps species differentiation remains to be seen, once the Florida Bay population is sequenced.

While we do not yet have enough information to assign any sort of host specificity to certain POE diatom taxa, we have enough DNA sequence data to suggest that some genetic differentiation among POE diatoms is occurring. While we do not know if the genetic distance between the Florida, Mediterranean and South African *Poulinea* strains is driven by speciation or intraspecific biogeography, they are genetically distinct. Data collected from loggerheads suggests little mixing between sea turtle individuals across ocean basins^38^, with the Mediterranean population being distinct from the northeast Atlantic one, which is then distinct from northwest Atlantic (including the Gulf of Mexico) population. Even within closer geographic boundaries, such as the western Atlantic, there is demonstrated genetic distance between POE strains (*C. caribeana* of Florida and the Bahamas; *Achnanthes elongata* of Florida and Georgia) in DNA sequence markers which are generally considered too conserved to show intraspecific variation in diatoms^39,40^.

The collection of molecular information from a larger number of POE diatom strains may reveal whether genetic diversity in epizoic diatoms reflects biogeographic, ecological, and behavioral patterns observed in the host animal populations. For example, it was demonstrated that sea turtle phylogeography is shaped by the sea turtle species thermal regime and habitat preference^41^. Provided the close relationship between epizoic diatoms and sea turtles holds up under the scrutiny of increased data sampling, it may be expected that POE diatoms associated with the cold-tolerant leatherbacks, which are able to use the southwestern corridors to migrate across the oceans, will be characterized by lower genetic diversity than diatom taxa growing on tropical species such as green turtles, hawksbills, and olive ridley sea turtles, whose Atlantic and Indo-Pacific populations appear to be genetically distinct^42^. This knowledge may significantly advance our understanding about evolutionary relationships between diatoms and their animal hosts as well as shed more light on the mechanistic processes of divergence and adaptive evolution of diatoms and other marine microbes.

This study lays the groundwork for biodiversity and biogeographical work in marine epibioses by starting the development of a database of DNA sequence data from 16 of the known POE diatom species for sea turtles and manatees. These sequences will also be useful in not only identifying more POE taxa, but searching for potential refugia of these taxa in non-epizoic habitats. Large areas of the world’s marine shallow benthic environment are poorly studied for diatoms, and therefore we cannot exclude the possibility that the POE taxa do exist outside of epizoic habitats. Even in localities that are relatively well-studied for benthic diatoms, variation in the composition and relative abundance in an assemblage due to substrate specificity and seasonality make the assembly of an exhaustive diatom flora extremely difficult. Environmental DNA surveys, such as metabarcoding, have an advantage over microscope-based surveys with regards to relatively small-sized taxa. Based on the molecular phylogeny of the *Tripterion* complex, it is easy to see how these taxa might have remained undetected in a bioinformatic summary of OTUs by sequence similarity, as there is significant genetic difference between the *Tripterion* complex and the only other sequenced representatives of the Rhoicospheniaceae—the freshwater taxon *Rhoicosphenia abbreviata* (C.Agardh) Lange-Bertalot. In fact, there are no morphological characters exclusive to the taxa in the molecular clade containing *Tursiocola* and the *Tripterion* complex that would cause a diatomist to expect a close match in sequence identity to the POE taxa. With curated sequence data now available for the most common POE taxa, we may find evidence for their occurrence in non-epizoic habitats through eDNA studies.

One of the stated goals of this study was to generate additional DNA sequence data from POE diatom taxa on sea turtles and sirenians. This goal was greatly aided by our ability to culture many of these POE diatoms away from their hosts, which raises several questions about the ecological requirements and adaptations of epizoic diatoms. The isolated strains of POE diatoms, which can be maintained in artificial conditions and without the animal hosts, provide opportunities to further study the molecular, genomic and physiological nature of the unique relationship between the diatoms and marine megafauna in a laboratory setting. For example, we can examine how different species may be affected by different conditions or possess specific adaptations to epizoic lifestyle. It is possible that some trade-off in obtaining those adaptations makes the POE taxa less competitive in non-epizoic benthic environments. We know little about the extent to which the microbes associated with the diatom (“phycosphere”) might affect the competitive ability of diatoms, and/or whether the phycosphere may itself manufacture some critical compound only in an epizoic community. Since all cultured POE diatoms were maintained as non-axenic cultures, it is yet unclear what role the bacterial strains played in the development and survival of the targeted diatom species and whether the long-term maintenance of axenic POE strains would be feasible. Future studies may also determine the number of evolutionary leaps to the epizoic habitat and the number of host switches, shedding more light on the co-evolution of diatom-animal relationships.

## Methods

### Cultures and microscopy

Diatoms were collected from the skin of West Indian manatees and the skin and carapace of six species of sea turtles (see Table 1 for details). These collections were made following the protocol outlined by Pinou et al.^43^. Wild sea turtles were either sampled on nesting beaches after oviposition (as to not disturb the nesting process) or from turtles captured in water via a rodeo method^44^. The seven sea turtles resident at the uShaka Sea World in Durban (South Africa) were sampled during feeding. The Adriatic Sea turtles were sampled upon arrival to the rescue center after being caught accidentally during trawling (Iracus) or during rehabilitation in an outdoor pool with freely circulating seawater (Lunga). Manatees were sampled during annual health assessments conducted by the USGS Sirenia Project.

Individual diatom cells were isolated by micropipette into sterile f/2 culture medium^45^ with a salinity matching that of the collection area. Strains isolated from the Bahamas, and the US were maintained under natural light in a north-facing window at UT Austin at room temperature (between 20 and 24 °C). South African strains were lit by natural light from a south-facing window and maintained at a temperature of 20–24 °C at the Unit of Environmental Sciences and Management in Potchefstroom. The strains isolated from the Adriatic were grown at 18–20 °C at 7–10 μmol m^2^ s^−1^, 12:12 (light:dark) cycle .In the case of non-photosynthetic taxa (like some *Tursiocola* species), individual cells were documented by light micrograph (“photovouchered”) and isolated into WGA whole-genome amplification cocktail^25^.

Cultures were harvested into separate pellets for microscopy preparation and DNA sequencing. Pellets for microscopy were cleaned with hydrogen peroxide and nitric acid, rinsed to neutral pH and dried onto 22 × 22 mm and 12 mm coverslips for light microscopy (LM) and scanning electron microscopy (SEM), respectively. Permanent mounts for the LM slides were made with Naphrax® mounting medium (Brunel Microscopes, www.brunelmicroscopessecure.co.uk) and micrographs were taken with a Zeiss Axioskop. Coverslips for SEM were coated with iridium by a Cressington 208 Bench Top Sputter Coater (Cressington Scientific Instruments, Watford, UK) and micrographs taken with a Zeiss SUPRA 40 VP scanning electron microscope (Carl Zeiss Microscopy, Thornwood, NY, USA). Additional micrographs of the strains are available from the authors.

### DNA isolation, amplification and sequencing

Pellets for DNA sequencing were extracted using the DNeasy Plant Minikit, with an extra 45 s incubation in a Beadbeater (Biospec Products, Bartlesville, OK, USA) with 1.0 mm glass pellets for colony and frustule disruption. The nuclear-encoded ribosomal SSU and chloroplast-encoded *rbc*L and *psb*C markers were amplified by PCR using the primers outlined in Theriot et al.^46^ in 25 µL reactions with 1–3 µL of template DNA, 0.5 µL of each primer, 0.25 µL of Taq polymerase, 12.5 µL of pre-mixed FailSafe Buffer E (Lucigen Corporation) and 8.25–10.25 µL of sterile water. PCR conditions were identical for *rbc*L and *psb*C: 94 °C for 3.5 min., 35 cycles of (94 °C for 30 s, 48 °C for 60 s., 72 °C for 2 min.), and a final extension at 72 °C for 15 min. PCR conditions for SSU were: 94 °C for 3.5 min., 35 cycles of (94 °C for 30 s., 51 °C for 60 s., 72 °C for 3 min.), and a final extension at 72 °C for 15 min. The amplicons were purified using an EXO-SAP protocol: a 3 µL of an EXO-SAP solution containing 0.5 µL of shrimp alkaline phosphatase, 0.25 µL of exonuclease I and 2.25 µL of sterile water were added to the PCR products and incubated at 37 °C for 30 min. followed by 80 °C for 15 min. Purified products were then sequenced on an ABI 3730 DNA Analyzers using BigDye Terminator v3.1 chemistry.

Sequence data were added to a dataset of raphid and araphid pennate diatoms, with *Asterionellopsis glacialis* used as an outgroup (see Table S1 for GenBank accession numbers). SSU data were aligned by the SSUalign program, using the covariance model outlined in Lobban et al.^47^. Data were initially partitioned by gene, by paired and unpaired sites in SSU secondary structure and codon position in *rbc*L and *psb*C. Model testing and grouping of partitions were performed by PartitionFinder 2^48^ using all nucleotide substitution models, linked branches, and rcluster search^49^ settings for trees inferred by RAxML 8^50^. The best model was chosen using the corrected Akaike information criterion (AICc). Maximum Likelihood and Bayesian Inference based phylogenies were inferred using IQ-TREE version 1.6.12 for Linux^51^ with partitioned models^52^ and multi-threaded MPI hybrid variant of ExaBayes version 1.5^53^, respectively. Nodal support for the maximum likelihood phylogeny was assessed using 1000 bootstrap replicates via IQ-TREE. ExaBayes analyses included four independent runs with two coupled chains where branch lengths were linked. Convergence parameters included an average deviation of split frequencies (ASDSF) of less than or equal to 5% with a minimum of 10,000,000 generations. Bayesian nodal support was assessed using posterior probabilities, with the first 25% of the trees removed as “burn-in”.

## Supplementary Information

Supplementary Figure S1.

Supplementary Figure S2.

Supplementary Table S1.

Supplementary Legends.

## Data Availability

DNA sequence data generated for this study are published on the NCBI GenBank online sequence depository under the accession numbers listed in Table S1. Additional micrographs and cleaned voucher material from the sequenced cultures are available from lead author MPA.

## Abbreviations

POE: Putatively obligate epizoic
SEM: Scanning electron microscope
bs: Bootstrap support
pp: Posterior probability

## References

[1] Zaneveld JR, McMinds R, Thurber RV (2017). Stress and stability: Applying the Anna Karenina principle to animal microbiomes. Nat. Microbiol..

[2] Trevelline BK, Fontaine SS, Hartup BK, Kohl KD (2019). Conservation biology needs a microbial renaissance: A call for the consideration of host-associated microbiota in wildlife management practices. Proc. R. Soc. B.

[3] Bennett AG (1920). On the occurrence of diatoms on the skin of whales. Proc. R. Soc. Lond. B.

[4] Denys L (1997). Morphology and taxonomy of epizoic diatoms (*Epiphalaina* and *Tursiocola*) on a sperm whale (*Physeter macrocephalus*) stranded on the coast of Belgium. Diatom. Res..

[5] Majewska R (2020). *Tursiocola neliana* sp. nov (Bacillariophyceae) epizoic on South African leatherback sea turtles (*Dermochelys coriacea*) and new observations on the genus Tursiocola. Phytotaxa.

[6] Majewska R, Kociolek J, Thomas E, De Stefano M, Santoro M, Bolanos F, Van de Vijver B (2015). *Chelonicola* and *Poulinea*, two new gomphonemoid genera living on marine turtles from Costa Rica. Phytotaxa.

[7] Majewska R, Van de Vijver B, Nasrolahi A, Ehsanpour M, Afkhami M, Bolaños F, Iamunno F, Santoro M, De Stefano M (2017). Shared epizoic taxa and differences in diatom community structure between green turtles (*Chelonia mydas*) from distant habitats. Microb Ecol..

[8] Majewska R, De Stefano M, Ector L, Bolaños F, Frankovich TA, Sullivan MJ, Ashworth MP, Van de Vijver B (2017). Two new epizoic *Achnanthes* species (Bacillariophyta) living on marine turtles from Costa Rica. Bot. Mar..

[9] Majewska R, De Stefano M, Van de Vijver B (2018). *Labellicula lecohuiana*, a new epizoic diatom species living on green turtles in Costa Rica. Nova Hedwig Beih..

[10] Majewska R, Ashworth MP, Lazo-Wasem E, Robinson NJ, Rojas L, Van de Vijver B, Pinou T (2018). *Craspedostauros alatus* sp. nov., a new diatom (Bacillariophyta) species found on museum sea turtle specimens. Diatom Res..

[11] Majewska R, Bosak S, Frankovich TA, Ashworth MP, Sullivan MJ, Robinson NJ, Lazo-Wasem EA, Pinou T, Nel R, Manning SR, Van de Vijver B (2019). Six new epibiotic *Proschkinia* (Bacillariophyta) species and new insights into the genus phylogeny. Eur. J. Phycol..

[12] Majewska R, Robert K, Van de Vijver B, Nel R (2020). A new species of *Lucanicum* (Cyclophorales, Bacillariophyta) associated with loggerhead sea turtles from South Africa. Bot. Lett..

[13] Frankovich TA, Sullivan MJ, Stacy NI (2015). *Tursiocola denysii* sp. Nov. (Bacillariophyta) from the neck skin of Loggerhead sea turtles (*Caretta caretta*). Phytotaxa.

[14] Frankovich TA, Ashworth MP, Sullivan MJ, Vesela J, Stacy NI (2016). *Medlinella amphoroidea* gen. et sp. Nov. (Bacillariophyta) from the neck skin of Loggerhead sea turtles (*Caretta caretta*). Phytotaxa.

[15] Riaux-Gobin C, Witkowski A, Kociolek JP, Ector L, Chevallier D, Compère P (2017). New epizoic diatom (Bacillariophyta) species from sea turtles in the Eastern Caribbean and South Pacific. Diatom Res..

[16] Riaux-Gobin C, Witkowski A, Chevallier D, Daniszewska-Kowalczyk G (2017). Two new *Tursiocola* species (Bacillariophyta) epizoic on green turtles (*Chelonia mydas*) in French Guiana and Eastern Caribbean. Fottea Olomouc.

[17] Riaux-Gobin C, Witkowski A, Kociolek JP, Chevallier D (2020). *Navicula dermochelycola* sp. Nov., presumably an exclusively epizoic diatom on sea turtles *Dermochelys coriacea* and *Lepidochelys olivacea* from French Guiana. Oceanol. Hydrobiol. Stud..

[18] Robert K, Bosak S, Van de Vijver B (2019). *Catenula exigua* sp. Nov., a new marine diatom (Bacillariophyta) species from the Adriatic Sea. Phytotaxa.

[19] Van de Vijver B, Bosak S (2019). *Planothidium kaetherobertianum*, a new marine diatom (Bacillariophyta) species from the Adriatic Sea. Phytotaxa.

[20] Robinson NJ, Majewska R, Lazo-Wasem EA, Nel R, Paladino FV, Rojas L, Zardus JD, Pinou T (2016). Epibiotic diatoms are universally present on all sea turtle species. PLoS ONE.

[21] Van de Vijver B, Robert K, Majewska R, Frankovich TA, Panagopolou A, Bosak S (2020). Diversity of diatom communities (Bacillariophyta) associated with loggerhead sea turtles. PLoS ONE.

[22] Van de Vijver B, Robert K, Witkowski A, Bosak S (2020). *Majewskaea* gen. nov. (Bacillariophyta), a new marine benthic diatom genus from the Adriatic Sea. Fottea.

[23] Majewska R (2021). *Nagumoea hydrophicola* sp. Nov. (Bacillariophyta), the first diatom species described from sea snakes. Diatom Res..

[24] Frankovich TA, Sullivan MJ, Stacey NI (2015). Three new species of *Tursiocola* (Bacillariophyta) from the skin of the West Indian manatee (*Trichechus manatus*). Phytotaxa.

[25] Frankovich TA, Ashworth MP, Sullivan MJ, Theriot EC, Stacy NI (2018). Epizoic and apochlorotic *Tursiocola* species (Bacillariophyta) from the skin of Florida manatees (*Trichechus manatus latirostris*). Protist.

[26] Azari M, Farjad Y, Nasrolahi A, De Stefano M, Ehsanpour M, Dobrestov S, Majewska R (2020). Diatoms on sea turtles and floating debris in the Persian Gulf (Western Asia). Phycologia.

[27] Majewska R, Goosen WE (2020). For better, for worse: Manatee-associated *Tursiocola* (Bacillariophyta) remain faithful to their host. J. Phycol..

[28] Smol JP, Stoermer EF (2010). The Diatoms: Applications for the Environmental and Earth Sciences.

[29] Rivera SF, Vasselon V, Ballorain K, Carpentier A, Wetzel CE, Ector L, Bouchez A, Rimet F (2018). DNA metabarcoding and microscopic analyses of sea turtles biofilms: Complementary to understand turtle behavior. PLoS ONE.

[30] Majewska R, Ashworth MP, Bosak S, Goosen WE, Nolte C, Filek K, Van de Vijver B, Taylor JC, Manning SR, Nel R (2021). On sea turtle-associated *Craspedostauros* with description of three novel species. J Phycol..

[31] Holmes RW (1985). The morphology of diatoms epizoic on cetaceans and their transfer from Cocconeis to two new genera, *Bennettella* and *Epipellis*. Br. Phycol. J..

[32] Woodworth KA, Frankovich TA, Freshwater DW (2019). *Melanothamnus maniticola* (Ceramiales, Rhodophyta): An epizoic species evolved for life on the West Indian Manatee. J. Phycol..

[33] Vitt LJ, Caldwell JP (2013). Herpetology: An Introductory Biology of Amphibians and Reptiles.

[34] Pitman LR, Durban JW, Joyce T, Fearnbach H, Panigada S, Lauriano G (2020). Skin in the game: Epidermal molt as a driver of long-distance migration in whales. Mar. Mamm. Sci..

[35] Pope DH, Berger LR (1973). Algal photosynthesis at increased hydrostatic pressure and constant pO_2_. Arch. Microbiol..

[36] Calcagno V, Jarne P, Loreau M, Mouquet N, David P (2017). Diversity spurs diversification in ecological communities. Nat. Commun..

[37] Robinson NJ, Pfaller JB (2021). Sea turtle epibiosis: Global patterns and knowledge gaps. Trends Evol. Ecol..

[38] Conant, T. A., Dutton, P. H., Eguchi, T., Epperly, S. P., Fahy, C. C., Godfrey, M. H., MacPherson, S. L., Possardt, E. E., Schroeder, B. A., Seminoff, J. A., Snover, M. L. Loggerhead sea turtle (*Caretta caretta*) 2009 status review under the US Endangered Species Act. In Report of the loggerhead biological review Team to the National Marine Fisheries Service. **222**, 1–230 (2009).

[39] Evans KM, Wortley AH, Mann DG (2007). An assessment of potential diatom ‘‘barcode’’ genes (cox1, rbcL, 18S and ITS rDNA) and their effectiveness in determining relationships in *Sellaphora* (Bacillariophyta). Protist.

[40] Hamsher SE, Evans KM, Mann DG, Poulíčková A, Saunders GW (2011). Barcoding diatoms: Exploring alternatives to COI-5P. Protist.

[41] Bowen BW, Karl SA (2007). Population genetics and phylogeography of sea turtles. Mol Ecol..

[42] Shanker K, Ramadevi J, Choudhury BC, Singh L, Aggarwal RK (2004). Phylogeography of olive ridley turtles (*Lepidochelys olivacea*) on the east coast of India: implications for conservation theory. Mol. Ecol..

[43] Pinou T, Domenech F, Lazo-Wasem EA, Majewska R, Pfaller JB, Zardus JD, Robinson NJ (2019). Standardizing sea turtle epibiont sampling: Outcomes of the epibiont workshop at the 37th International Sea Turtle Symposium. Mar. Turt. Newsl..

[44] Ehrhert L., Ogren L. H. Studies in foraging habitats: capturing and handling turtles. In *Research and management techniques for the conservation of sea turtles* (eds. Eckert, K. L., Bjorndal, K. A., Abreu-Grobois, F. A., Donnelly, M.). IUCN/SSC Marine Turtle Specialist Group. Publication No. 4. (1999).

[45] Guillard, R. R. Culture of phytoplankton for feeding marine invertebrates. In *Culture of Marine Invertebrate Animals* 29–60 (Springer, 1975).

[46] Theriot EC, Ashworth MP, Nakov T, Ruck E, Jansen RK (2015). Dissecting signal and noise in diatom chloroplast protein encoding genes with phylogenetic information profiling. Mol. Phylogenet. Evol..

[47] Lobban CS, Ashworth MP, Calaor JJ, Theriot EC (2019). Extreme diversity in fine-grained morphology reveals fourteen new species of conopeate *Nitzschia* (Bacillariophyta: Bacillariales). Phytotaxa..

[48] Lanfear R, Frandsen PB, Wright AM, Senfeld T, Calcott B (2017). PartitionFinder 2: New methods for selecting partitioned models of evolution for molecular and morphological phylogenetic analyses. Mol. Biol. Evol..

[49] Lanfear R, Calcott B, Kainer D, Mayer C, Stamatakis A (2014). Selecting optimal partitioning schemes for phylogenomic datasets. BMC Evol. Biol..

[50] Stamatakis A (2014). RAxML version 8: A tool for phylogenetic analysis and post-analysis of large phylogenies. Bioinformatics.

[51] Nguyen L-T, Schmidt HA, von Haeseler A, Minh BQ (2015). IQ-TREE: A fast and effective stochastic algorithm for estimating maximum likelihood phylogenies. Mol. Biol. Evol..

[52] Chernomor O, Von Haeseler A, Minh BQ (2016). Terrace aware data structure for phylogenomic inference from supermatrices. Syst. Biol..

[53] Aberer AJ, Kobert K, Stamatakis A (2014). ExaBayes: Massively parallel bayesian tree inference for the whole-genome Era. Mol. Biol. Evol..

