# Supplementary figures and images for: Cultivating epizoic diatoms provides insights into the evolution and ecology of both epibionts and hosts
Source: Sci Rep. 2022 Sep 6;12:15116. doi: 10.1038/s41598-022-19064-0 (PMC9448772; doi:10.1038/s41598-022-19064-0)

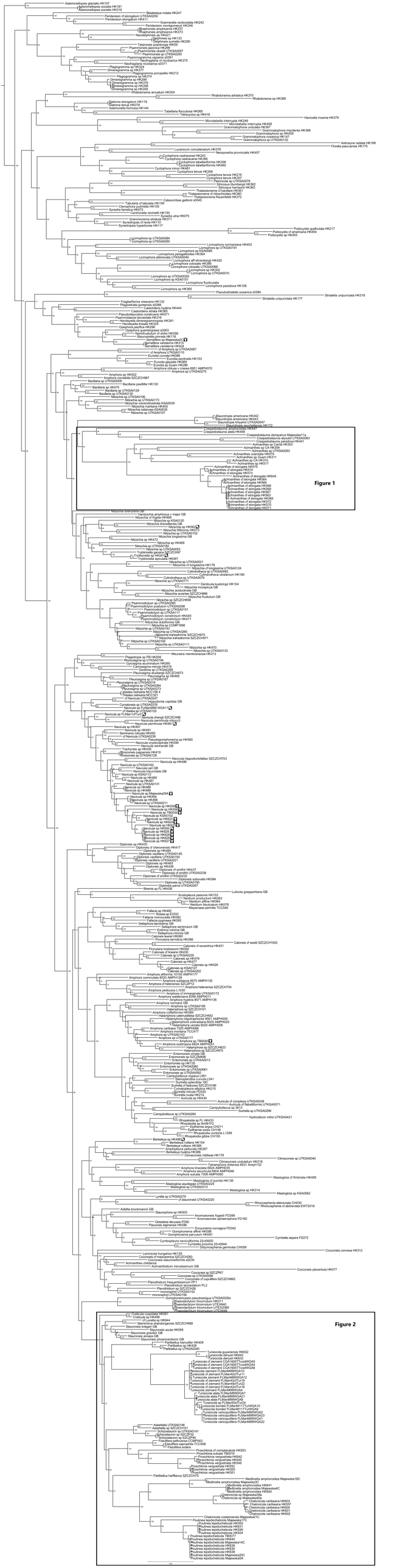

Supplement: Supplementary file 1 — Supplementary Figure S1. [file 41598_2022_19064_MOESM1_ESM.pdf]

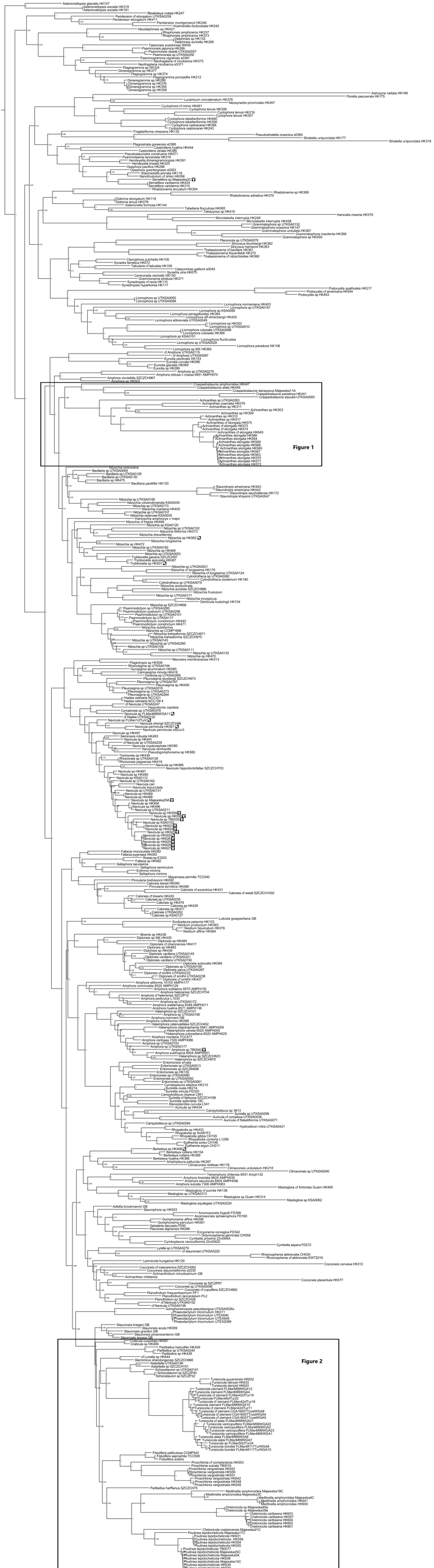

Supplement: Supplementary file 2 — Supplementary Figure S2. [file 41598_2022_19064_MOESM2_ESM.pdf]
