# Supplementary material for: Cultivating epizoic diatoms provides insights into the evolution and ecology of both epibionts and hosts
Source: Sci Rep. 2022 Sep 6;12:15116. doi: 10.1038/s41598-022-19064-0 (PMC9448772; doi:10.1038/s41598-022-19064-0)
Supplement: Supplementary file 3 — Supplementary Table S1. [file 41598_2022_19064_MOESM3_ESM.doc]

Table S1. Taxa, strain voucher ID and GenBank accession numbers for strains used in the DNA sequence data phylogenetic analysis. Collection site for sample of original strain isolation is also included (where known); in the case of cultures from public collections, the culture ID is provided in this column (UTEX = UTEX Culture Collection of Algae; NCMA = National Center for Marine Algae and Microbiota; CSIRO = Australian National Algae Culture Collection; MCC-NIES = Microbial Culture Collection at National Institute for Environmental Studies). Ingroup taxa (raphid pennates) provided first in the table; outgroup taxa (“araphid pennates”) follow after table break. Taxa are listed alphabetically. If species unknown, authority for genus is listed.

| Taxon | Strain Voucher | Collection Site  (Locality in paretheses) | GenBank Accession  (SSU, *rbc*L, *psb*C) |
| --- | --- | --- | --- |
| *Achnanthes chlidanos* M.H.Hohn & Hellerman |  |  | KJ658412, KJ658394, N/A |
| *Achnanthes coarctata* (Brébisson ex W. Smith) Grunow in Cleve & Grunow | HK079 | FD185 (UTEX) | HQ912594, HQ912458, HQ912287 |
| *Achnanthes elongata* Majewska & Van de Vijver | HK563 | Green Sea turtle (Turtle Hospital, Marathon, Florida) | N/A, MT432475, MT432494 |
| *Achnanthes elongata* Majewska & Van de Vijver | HK564 | Green Sea turtle (Turtle Hospital, Marathon, Florida) | MT441506, MT432476, MT432495 |
| *Achnanthes elongata* Majewska & Van de Vijver | HK565 | Green Sea turtle (Turtle Hospital, Marathon, Florida) | MT441507, MT432477, MT432496 |
| *Achnanthes elongata* Majewska & Van de Vijver | HK566 | Green Sea turtle (Turtle Hospital, Marathon, Florida) | MT441504, MT432473, MT432492 |
| *Achnanthes elongata* Majewska & Van de Vijver | HK567 | Green Sea turtle (Turtle Hospital, Marathon, Florida) | MT441505, MT432474, MT432493 |
| *Achnanthes elongata* Majewska & Van de Vijver | HK568 | Loggerhead turtle (Florida Bay, Florida) | MT441510, MT432481, MT432500 |
| *Achnanthes elongata* Majewska & Van de Vijver | HK569 | Loggerhead turtle (Florida Bay, Florida) | MT441511, MT432482, MT432501 |
| *Achnanthes elongata* Majewska & Van de Vijver | HK570 | Kemp’s ridley turtle (Georgia) | MT441508, MT432478, MT432497 |
| *Achnanthes elongata* Majewska & Van de Vijver | HK571 | Kemp’s ridley turtle (Georgia) | MT441509, MT432479, MT432498 |
| *Achnanthes elongata* Majewska & Van de Vijver | HK572 | Kemp’s ridley turtle (Georgia) | N/A, MT432480, MT432499 |
| *Achnanthes* cf *elongata* Majewska & Van de Vijver | HK573 | West Indian Manatee (Ft. Lauderdale, Florida) | MT441502, MT432471, MT432490 |
| *Achnanthes* cf *elongata* Majewska & Van de Vijver | HK574 | West Indian Manatee (Ft. Lauderdale, Florida) | MT441501, MT432470, MT432489 |
| *Achnanthes* cf *elongata* Majewska & Van de Vijver | HK575 | West Indian Manatee (Georgia) | MT441503, MT432472, MT432491 |
| *Achnanthes* cf *elongata* Majewska & Van de Vijver | HK645 | Green Sea turtle (Florida Bay, Florida) |  |
| *Achnanthes* sp*.* Bory | HK303 | SanNicholas1 (San Nicholas, Canary Islands) | KC309473, KC309545, KC309617 |
| *Achnanthes* sp. Bory | HK309 | ECT3883 (Rainbow Harbor, Long Beach, California) | KC309474, KC309546, KC309618 |
| *Achnanthes* sp. Bory | HK310 | ECT3911 (Long Beach, California) | KC309475, KC309547, KC309619 |
| *Achnanthes* sp. Bory | HK311 | ECT3684 (Achang Reef, Guam) | KC309476, KC309548, KC309620 |
| *Achnanthes* sp. Bory | HK517 | Azo42 (Azores) | MH063437, MH064054, MH063967 |
| *Achnanthes* sp. Bory | UTKSA0263 | KSA2015-16 (Al-Nawras, Jeddah, Saudi Arabia) | MH063438, MH064055, N/A |
| *Achnanthidium minutissimum* (Kützing) Czarnecki |  |  | AM502032, AM710499, N/A |
| *Adlafia brockmannii* (Hustedt) Bruder & Hinz |  |  | AM502020, AM710487, N/A |
| *Amphipleura pellucida* Kützing | HK287 | ECT3568 (Lake Travis, Texas) | KC309477, KC309549, KC309621 |
| *Amphora aliformis* J.G. Stepanek, S. Mayama & Kociolek |  | AMPH177 | KP229525, KP229546, KP229548 |
| *Amphora caribaea* Wachnicka & E.E.Gaiser |  | AMPH086 | KJ463428, KJ463458, KJ463488 |
| *Amphora commutata* Grunow |  | AMPH126 | KP229526, KP229547, KP229549 |
| *Amphora* cf *immarginata* Nagumo | UTKSA0172 | KSA2015-37 (Rabigh, Saudi Arabia) | MH063439, MH064056, MH063968 |
| *Amphora helenensis* M.H. Giffen |  | SZCZCH704 | KT943649, KT943672, KT943709 |
| *Amphora* cf *helenensis* M.H. Giffen |  | SZCZP12 | KU179126, KU179113, KU179140 |
| *Amphora hyalina* Kützing |  | AMPH136 | KJ463432, KJ463462, KJ463492 |
| *Amphora lineolata* Ehrenberg |  | AMPH035 | KJ463435, KJ463465, KJ463495 |
| *Amphora obtusa* Gregory | UTKSA0275 | KSA2015-37 (Rabigh, Saudi Arabia) | MH063440, MH064057, N/A |
| *Amphora obtusa v crassa* |  | AMPH070 | KJ463436, KJ463466, KJ463496 |
| *Amphora pediculus (Kützing) Grunow* |  | L1030 (UTEX) | HQ912417, HQ912403, HQ912389 |
| *Amphora securicula* H. Peragallo & M. Peragallo |  | AMPH046 | KJ463440, KJ463470, KJ463500 |
| *Amphora* sp*.* Ehrenberg ex Kützing | HK502 | PackaryChannelSediment (Mustang Island, Texas) | MH017634, MH064058, MH063969 |
| *Amphora* sp*.* Ehrenberg ex Kützing |  | PMFTB0040 |  |
| *Amphora* sp*.* Ehrenberg ex Kützing | UTKSA0087 | SA12 (Markaz Al Shoaibah, Saudi Arabia) | MH063441, MH064059, MH063970 |
| *Amphora* sp*.* Ehrenberg ex Kützing | UTKSA0115 | KSA2015-27 (Markaz Al Shoaibah, Saudi Arabia) | MH063442, MH064060, N/A |
| *Amphora* sp*.* Ehrenberg ex Kützing | UTKSA0153 | KSA2015-37 (Rabigh, Saudi Arabia) | MH063443, MH064061, MH063971 |
| *Amphora* sp*.* Ehrenberg ex Kützing | UTKSA0177 | KSA2015-41 (Rabigh, Saudi Arabia) | MH063444, MH064062, MH063972 |
| *Amphora sublaevis* Hustedt |  | AMPH135 | KJ463444, KJ463474, KJ463504 |
| *Amphora subtropica* Wachnicka & E.E.Gaiser |  | AMPH051 | KJ463445, KJ463475, KJ463505 |
| *Amphora sulcata* Brébisson |  | AMPH083 | KJ463446, KJ463476, KJ463506 |
| *Amphora vixvisibilis* Li & Witkowski |  | SZCZCH967 | KT943648, KT943670, KT943706 |
| *Amphora waldeniana* J.G. Stepanek & Kociolek |  | AMPH011 | KJ463447, KJ463477, KJ463507 |
| *Anomoeoneis fogedii* Reimer |  | FD399 (UTEX) | KJ011610, KJ011793, N/A |
| *Anomoeoneis sphaerophora* Pfitzer |  | FD160 (UTEX) | KJ011612, KJ011795, N/A |
| *Astartiella almalikii* J.S.M. Sabir, Ashworth & Górecka | UTKSA0146 | KSA2015-11 (Bhadur Resort, Saudi Arabia) | MH063445, MH064063, MH063973 |
| *Astartiella chunlianlii* Witkowski, Ashworth, Górecka & Dąbek |  | SZCZCH151 | N/A, KT943613, KT943624 |
| *Auricula* sp*.* Castracane | HK434 | 21IV14-4D (Rabbit Key Basin, Florida) | KX981842, KX981810, KX981789 |
| *Auricula* cf *complexa* (Gregory) Cleve | UTKSA0038 | SA12 (Markaz Al Shoaibah, Saudi Arabia) | MH063446, MH064064, MH063974 |
| *Auricula* cf *flabelliformis* M. Voigt | UTKSA0071 | SA12 (Markaz Al Shoaibah, Saudi Arabia) | MH063447, MH064065, MH063975 |
| *Bacillaria paxillifer* (O. F. Müller) T. Marsson | HK130 | FD468 (UTEX) | HQ912627, HQ912491, HQ912320 |
| *Bacillaria* sp*.* J.F.Gmelin | HK475 | GU44BK-1 (Gab Gab Beach, Guam) | MH063448, MH064066, MH063976 |
| *Bacillaria* sp*.* J.F.Gmelin | UTKSA0009 | SA27 (Jeddah, Saudi Arabia) | MH063449, MH064067, MH063977 |
| *Bacillaria* sp*.* J.F.Gmelin | UTKSA0129 | KSA2015-9 (Bhadur Resort, Saudi Arabia) | MH063450, MH064068, MH063978 |
| *Bacillaria* sp*.* J.F.Gmelin | UTKSA0130 | KSA2015-9 (Bhadur Resort, Saudi Arabia) | MH063451, MH064069, MH063979 |
| *Berkeleya hyalina* (F.E.Round & M.E.Brooks) E.J.Cox | HK388 | ECT3614 (La Jolla, California) | KJ577847, KJ577882, KJ577917 |
| *Berkeleya rutilans* (Trentepohl ex Roth) Grunow | HK154 | ECT3616 (Laguna Beach, California) | HQ912637, HQ912501, HQ912330 |
| *Berkeleya rutilans* (Trentepohl ex Roth) Grunow | HK389 | ECT3602 (Bolinas, California) | KJ577848, KJ577883, KJ577918 |
| *Berkeleya* sp. Greville | HK498 | West Indian Manatee (Georgia) |  |
| *Biremis* sp*.* D.G. Mann & E.J. Cox | HK438 | 21IV14-2A (Duck Key, Florida) | KX981835, KX981811, N/A |
| *Caloneis lewisii* Patrick | HK060 | FD54 (UTEX) | HQ912580, HQ912444, HQ912273 |
| *Caloneis* sp*.* P.T. Cleve | KSA0127 | SA12 (Markaz Al Shoaibah, Saudi Arabia) | KU179135, KU179125, N/A |
| *Caloneis* sp*.* P.T. Cleve | HK429 | SantaRosa cor.green (Costa Rica) | KU179134, KU179123, N/A |
| *Caloneis* cf *linearis* (Cleve) Boyer | HK430 | 21IV14-3A (Captain’s Key, Florida) | KU179132, KU179119, KU179146 |
| *Caloneis* cf *excentrica* (Grunow) Boyer | HK431 | 21IV14-2A (Duck Key, Florida) | KU179130, KU179117, KU179144 |
| *Caloneis* sp*.* P.T. Cleve | HK477 | GU7Y-4 (University of Guam Marine Laboratories, Guam) | MH063453, MH064071, MH063981 |
| *Caloneis* sp*.* P.T. Cleve | HK479 | GU52V-2 (Outhouse Beach, Guam) | N/A, MH064072, MH063982 |
| *Caloneis* sp*.* P.T. Cleve | UTKSA0235 | KSA2015-37 (Rabigh, Saudi Arabia) | MH063454, MH064073, MH063983 |
| *Caloneis* sp*.* P.T. Cleve | UTKSA0252 | KSA2015-42 (Rabigh, Saudi Arabia) | MH063455, MH064074, MH063984 |
| *Caloneis* cf *westii* (W. Smith) Hendey |  | SZCZCH1002 | KT943628, KT943654, KT943687 |
| *Campylodiscus clypeus* (Ehrenberg) Kützing |  | L951 (UTEX) | HQ912412, HQ912398, HQ912384 |
| *Campylodiscus* sp*.* Ehrenberg ex Kützing |  | ECT3613 (Tomales Bay, California) | HQ912413, HQ912399, HQ912385 |
| *Campylodiscus* sp*.* Ehrenberg ex Kützing | UTKSA0284 | KSA2015-29 (Markaz Al Shoaibah, Saudi Arabia) | MH063456, MH064075, N/A |
| *Carinasigma minuta* (Donkin) G. Reid | HK418 | GU7X-6 (University of Guam Marine Lab, Guam) | KX981841, KX981812, KX981790 |
| *Chelonicola caribeana* Riaux-Gobin, Witkowski, Ector & D.Chevallier | HK557 | Loggerhead turtle (Florida Bay, Florida) |  |
| *Chelonicola caribeana* Riaux-Gobin, Witkowski, Ector & D.Chevallier | HK601 | Green sea turtle (Bahamas) |  |
| *Chelonicola caribeana* Riaux-Gobin, Witkowski, Ector & D.Chevallier | HK602 | Green sea turtle (Bahamas) |  |
| *Chelonicola caribeana* Riaux-Gobin, Witkowski, Ector & D.Chevallier | HK603 | Loggerhead turtle (Florida Bay, Florida) |  |
| *Chelonicola caribeana* Riaux-Gobin, Witkowski, Ector & D.Chevallier | HK604 | Loggerhead turtle (Florida Bay, Florida) |  |
| *Chelonicola* *costaricensis*. Majewska, De Stefano & Van de Vijver |  | Majewska17C |  |
| *Chelonicola* sp. Majewska, De Stefano & Van de Vijver |  | Majewska39A |  |
| *Chelonicola* sp. Majewska, De Stefano & Van de Vijver |  | Majewska40A |  |
| *Climaconeis riddleae* Prasad | HK178 | ECT3724 (Umatac Bay, Guam) | HQ912644, HQ912508, HQ912337 |
| *Climaconeis* sp*.* Grunow | UTKSA0040 | SA26 (Jeddah, Saudi Arabia) | KX981836, KX981813, N/A |
| *Climaconeis undulata* (Meister) Lobban et al. | HK218 | ECT3743 (Talofofo Bay, Guam) | KC309478, KC309550, N/A |
| *Cocconeis* cf *cupulifera* Riaux-Gobin, Romero, Compère & Al-Handal |  | SZCZCH662 | N/A, KT943680, KT943718 |
| *Cocconeis* cf *mascarenica* Riaux-Gobin & Compère |  | SZCZCH283 | N/A, KT943679, KT943717 |
| *Cocconeis placentula* Ehrenberg | HK077 | FD23 (UTEX) | HQ912592, HQ912456, HQ912285 |
| *Cocconeis stauroneiformis* (W. Smith) H. Okuna |  | s0230 | AB430614, AB430694, N/A |
| *Cocconeis* sp. Ehrenberg | UTKSA0056 | SA28 (Jeddah, Saudi Arabia) | KU179133, KU179120, KU179147 |
| *Cocconeis convexa* M.H. Giffen | HK312 | ECT3901 (Channel #5, US-1, Florida) | KC309479, KC309551, KC309622 |
| *Cocconeis* sp. Ehrenberg |  | SZCZP67 | KT943600, KT943614, KT943625 |
| *Craspedostauros alatus* Majewska & Ashworth | HK448 | CCMP1120 (NCMA) | KX981860, KX981817, KX981793 |
| *Craspedostauros alyoubii* J. Sabir & Ashworth | UTKSA0083 | SA18 (Duba, Saudi Arabia) | KX981857, KX981814, KX981791 |
| *Craspedostauros amphoroides* (Grunow) Cox | HK447 | CCMP797 (NCMA) | KX981859, KX981815, N/A |
| *Craspedostauros danayanus* Majewska & Ashworth | HK637 | Leatherback turtle (Mabibi Beach, South Africa) | N/A, MT432485, MT432505 |
| *Craspedostauros mcewanii* Majewska & Ashworth | HK634 | RM July19 Calypso  Green Sea Turtle (Durban, South Africa) | N/A, MT432486, MT432505 |
| *Craspedostauros mcewanii* Majewska & Ashworth | HK635 | RM July19 Calypso  Green Sea Turtle (Durban, South Africa) | N/A, MT432487, N/A |
| *Craspedostauros mcewanii* Majewska & Ashworth | HK636 | RM July19 Calypso  Green Sea Turtle (Durban, South Africa) | N/A, MT432488, N/A |
| *Craspedostauros paradoxus* Ashworth & Lobban | HK441 | GU44BK-1 (Gab Gab Beach, Guam, USA) | KX981858, KX981816, KX981792 |
| *Craticula cuspidata* (Kützing) Mann | HK061 | FD35 (UTEX) | HQ912581, HQ912445, HQ912274 |
| *Craticula* sp. Grunow | HK499 | West Indian Manatee (Crystal River, Florida) |  |
| *Cylindrotheca closterium* (Ehrenberg) Reimann & Lewin | HK180 | CCMP1855 (NCMA) | HQ912645, HQ912509, HQ912338 |
| *Cylindrotheca* sp*.*  Rabenhorst | UTKSA0079 | SA12 (Markaz Al Shoaibah, Saudi Arabia) | KX981848, KX981826, KX981801 |
| *Cylindrotheca* sp*.*  Rabenhorst | UTKSA0082 | SA18 (Duba, Saudi Arabia) | KX981847, KX981827, KX981802 |
| *Cymatoneis* sp*.* Cleve | UTKSA0378 | KSA2016-3 (Bhadur Resort, Saudi Arabia) | MH063457, MH064076, MH063985 |
| *Cymatopleura elliptica* (Brebisson ex Kutzing) W. Smith | HK215 | L1333 (UTEX) | HQ912659, HQ912523, HQ912352 |
| *Cymbella aspera* (Ehrenberg) Cleve |  | FD272 (UTEX) | KJ011615, KJ011797, N/A |
| *Cymbella proxima* Reimer |  |  | AM502017, AM710484, N/A |
| *Cymbopleura naviculiformis* (Auerswald ex Heiberg) Krammer |  |  | AM502004, AM710471, N/A |
| *Denticula kuetzingii* Grunow | HK104 | FD135 (UTEX) | HQ912610, HQ912474, HQ912303 |
| *Didymosphenia geminata* (Lyngbye) M. Schmidt |  | CH058 | KJ011636, KJ011819, N/A |
| *Diploneis* cf *cheronensis* (Grunow) Cleve | HK417 | GU44AY-6 (Gab Gab Beach, Guam) | MH017637, MH064077, MH063986 |
| *Diploneis parca* (Schmidt in Schmidt et al.) Boyer | UTKSA0267 | KSA2015-49 (Duba, Saudi Arabia) | MH063458, MH064078, MH063987 |
| *Diploneis* cf *smithii* (Brébisson in W. Smith) P.T. Cleve | HK437 | GU44AY-6 (Gab Gab Beach, Guam) | KX981837, KX981818, KX981794 |
| *Diploneis* cf *smithii* (Brébisson in W. Smith) P.T. Cleve | UTKSA0232 | KSA0215-30 (Markaz Al Shoaibah, Saudi Arabia) | MH063459, MH064079, N/A |
| *Diploneis* cf *smithii* (Brébisson in W. Smith) P.T. Cleve | UTKSA0238 | KSA2015-37 (Rabigh, Saudi Arabia) | MH063460, MH064080, MH063988 |
| *Diploneis* sp*.* (Ehrenberg) P.T. Cleve | HK435 | Coz-4 (Cozumel, Mexico) | KX981839, KX981819, KX981795 |
| *Diploneis* sp*.* (Ehrenberg) P.T. Cleve | HK436 | Coz-4 (Cozumel, Mexico) | KX981838, KX981820, KX981796 |
| *Diploneis* sp*.* (Ehrenberg) P.T. Cleve | HK483 | 21IV14-2A (Duck Key, Florida) | MH017638, MH064081, MH063989 |
| *Diploneis* sp*.* (Ehrenberg) P.T. Cleve | HK484 | PackaryChannelPlank (Mustang Island, Texas) | MH017639, MH064082, MH063990 |
| *Diploneis* sp*.* (Ehrenberg) P.T. Cleve | UTKSA0190 | KSA2015-14 (Bhadur Resort, Saudi Arabia) | MH063461, MH064083, MH063991 |
| *Diploneis subovalis* Cleve | HK084 | FD282 (UTEX) | HQ912597, HQ912461, HQ912290 |
| *Diploneis vacillans* (A.W.F. Schmidt) Cleve | UTKSA0145 | KSA2015-11 (Bhadur Resort, Saudi Arabia) | MH063462, MH064084, MH063992 |
| *Diploneis vacillans* (A.W.F. Schmidt) Cleve | UTKSA0150 | KSA2015-37 (Rabigh, Saudi Arabia) | N/A, MH064085, MH063993 |
| *Diploneis vacillans* (A.W.F. Schmidt) Cleve | UTKSA0221 | KSA2015-7 (Bhadur Resort, Saudi Arabia) | N/A, MH064086, MH063994 |
| *Donkinia* sp*.* Ralfs | UTKSA0269 | KSA2015-37 (Rabigh, Saudi Arabia) | MH063463, MH064087, MH063995 |
| *Encyonema norvegica* (Grunow) Mayer |  | FD342 (UTEX) | KJ011643, KJ011826, N/A |
| *Entomoneis ornata* (Bailey) Reimer |  |  | HQ912411, HQ912397, HQ912383 |
| *Entomoneis* sp*.* Ehrenberg | HK 135 | CS782 (CSIRO) | HQ912631, HQ912495, HQ912324 |
| *Entomoneis* sp*.* Ehrenberg |  | SZCZM496 | KT943630, KT943656, KT943689 |
| *Entomoneis* sp*.* Ehrenberg | UTKSA0013 | SA12 (Markaz Al Shoaibah, Saudi Arabia) | N/A, MH064088, MH063996 |
| *Entomoneis* sp*.* Ehrenberg | UTKSA0061 | SA18 (Duba, Saudi Arabia) | MH063464, MH064089, MH063997 |
| *Entomoneis* sp*.* Ehrenberg | UTKSA0080 | SA12 (Markaz Al Shoaibah, Saudi Arabia) | MH063465, MH064090, MH063998 |
| *Entomoneis* sp*.* Ehrenberg | UTKSA0092 | SA18 (Duba, Saudi Arabia) | MH063466, MH064091, MH063999 |
| *Eolimna minima* (Grunow in Van Heurck) H. Lange-Bertalot |  |  | AM501962, AM710427, N/A |
| *Epithemia argus* (Ehrenberg) Kützing |  | CH211 | HQ912408, HQ912394, HQ912380 |
| *Epithemia sorex*  Kützing |  | CH148 | HQ912409, HQ912395, HQ912381 |
| *Eunotia curvata* Lagerstedt | HK086 | FD412 (UTEX) | HQ912599, HQ912463, HQ912292 |
| *Eunotia glacialis* Meister | HK069 | FD46 (UTEX) | HQ912586, HQ912450, HQ912279 |
| *Eunotia pectinalis* (Kützing) Rabenhorst | HK153 | NIES461 (MCC-NIES) | HQ912636, HQ912500, HQ912329 |
| *Eunotia* sp*.* Ehrenberg | HK286 | ECT3676 (Tinago River, Guam) | KC309480, KC309552, KC309623 |
| *Fallacia monoculata* (Hustedt) Mann | HK082 | FD254 (UTEX) | HQ912596, HQ912460, HQ912289 |
| *Fallacia pygmaea* (Kützing) Stickle & Mann | HK093 | FD294 (UTEX) | HQ912605, HQ912469, HQ912298 |
| *Fallacia* sp*.* Stickle & D.G. Mann | HK482 | GU52X-3 (Outhouse Beach, Guam) | MH063467, MH064092, MH064000 |
| *Fistulifera pelliculosa* (Brebisson) Lange-Bertalot |  |  | AY485454, HQ337547, N/A |
| *Fistulifera saprophila* (Lange-Bertalot & Bonik) Lange-Bertalot |  |  | KC736618, KC736593, N/A |
| *Fistulifera solaris* S.Mayama, M.Matsumoto, K.Nemoto & T.Tanaka |  |  | N/A, |
| *Geissleria decussis* (Østrup) Lange-Bertalot & Metzeltin |  | FD050 (UTEX) | KJ011647, KJ011830, N/A |
| *Gomphonema affine* Kützing | HK098 | FD173 (UTEX) | HQ912608, HQ912472, HQ912301 |
| *Gomphonema parvulum* (Kützing) Kützing | HK081 | FD241 (UTEX) | HQ912595, HQ912459, HQ912288 |
| *Gomphonemopsis* cf *pseudoexigua* (Simonsen) Medlin | UTKSA0026x | SA18 (Duba, Saudi Arabia) | MH063471, MH064098, MH064005 |
| *Gyrosigma acuminatum* (Kützing) Rabenhorst | HK085 | FD317 (UTEX) | HQ912598, HQ912462, HQ912291 |
| *Halamphora catenulafalsa* Witkowski & Ch. Li |  | SZCZCH452 | KT943646, KT943669, KT943704 |
| *Halamphora coffeaeformis* (Agardh) Levkov | HK089 | FD75 (UTEX) | HQ912602, HQ912466, HQ912295 |
| *Halamphora* cf *costata* (Smith) Levkov | UTKSA0195 | KSA2015-22 (Markaz Al Shoaibah, Saudi Arabia) | MH063468, MH064093, MH064001 |
| *Halamphora coloradiana* J.G. Stepanek & J.P. Kociolek |  | AMPH025 | KJ463450, KJ463480, KJ463510 |
| *Halamphora montana* (Krasske) Levkov |  | TCC477 | KC736615, KC736590, N/A |
| *Halamphora normanii* (Rabenhorst) Levkov |  |  | AM501958, AM710424, N/A |
| *Halamphora oligotraphenta* (Lange-Bertalot) Levkov |  | AMPH009 | KJ463451, KJ463481, KJ463511 |
| *Halamphora* sp*.* (Cleve) Levkov |  | SZCZCH101 | KT943645, KT943682, KT943703 |
| *Halamphora* sp*.* (Cleve) Levkov |  | SZCZCH623 | KT943647, KT943684, KT943705 |
| *Halamphora* sp*.* (Cleve) Levkov |  | SZCZCH975 | KT943650, KT943673, KT943710 |
| *Halamphora veneta* (Kützing) Levkov |  | AMPH005 | KJ463452, KJ463482, KJ463512 |
| *Hantzschia amphioxys v. major* Grunow in Van Heurck |  |  | HQ912404, HQ912390, HQ912376 |
| *Haslea* cf *howeana* (Hagelstein) Giffen | HK494 | GU7Y-4 (University of Guam Marine Labs, Guam) | N/A, MH040268, MH040241 |
| *Haslea* cf *howeana* (Hagelstein) Giffen | HK496 | PR6 (San Juan, Puerto Rico) | MH017640, MH040269, MH040242 |
| *Haslea ostrearia* (Gaillon) Simonsen |  | NCC158.4 | N/A, HF563525, HF558667 |
| *Haslea ostrearia* (Gaillon) Simonsen |  | NCC321 | N/A, HF563527, HF558669 |
| cf *Haslea sp.* Simonsen | UTKSA0122 | KSA2015-30 (Markaz Al Shoaibah, Saudi Arabia) | N/A, MH064096, MH064004 |
| *Hippodonta capitata* (Ehrenberg) Lange-Bertalot, Metzeltin & Witkowski |  |  | AM501966, AM710432, N/A |
| *Hydrosilicon mitra* Brun | UTKSA0421 | KSA2015-37 (Rabigh, Saudi Arabia) | MH063470, MH064097, N/A |
| *Lemnicola hungarica* (Grunow) Round | HK129 | FD456 (UTEX) | HQ912626, HQ912490, HQ912319 |
| cf *Lunella* sp. P. Snoeijs | HK644 |  |  |
| *Luticola goeppertiana* (Bleisch) D.G.Mann ex J.Rarick, S.Wu, S.S.Lee & Edlund |  |  | AM501967, AM710433, N/A |
| *Lyrella hennedyi* (W. Smith) Stickle & Mann | UTKSA0279 | KSA2015-5 (Bhadur Resort, Saudi Arabia) | MH063472, MH064099, |
| *Mastogloia aquilegiae* Grunow in Moller | UTKSA0224 | KSA2015-49 (Duba, Saudi Arabia) | N/A, MH064100, MH064007 |
| *Mastogloia fimbriata* (T. Brightwell) Grunow | HK485 | GU52X-1 (Outhouse Beach, Guam) | MH040321, MH040270, MH040243 |
| *Mastogloia* cf *pumila* (Grunow) Cleve | HK136 | 29X07-6B (Mustang Island, Texas) | HQ912632, HQ912496, HQ912325 |
| *Mastogloia* sp*.* Thwaites in W. Smith | HK314 | ECT3762 (Taeleyag Beach, Guam) | KC309481, KC309553, N/A |
| *Mastogloia* sp*.* Thwaites in W. Smith | KSA0062 | SA17 (Duba, Saudi Arabia) | MH063473, MH064101, MH064008 |
| *Mastogloia* sp*.* Thwaites in W. Smith | UTKSA0313 | KSA0216-44 (Markaz Al Shoaibah, Saudi Arabia) | MH063474, MH064102, MH064009 |
| *Mayamea perimitis* (Hustedt) K. Bruder & L.K. Medlin |  | TCC540 | KC736630, KC736600, N/A |
| *Medlinella amphoroidea* Frankovich, M.P.Ashworth & M.J.Sullivan | HK600 | Loggerhead turtle (South Africa) |  |
| *Medlinella amphoroidea* Frankovich, M.P.Ashworth & M.J.Sullivan | HK641 | Majewska9C-reisolation |  |
| *Medlinella amphoroidea* Frankovich, M.P.Ashworth & M.J.Sullivan |  | Majewska3C |  |
| *Medlinella amphoroidea* Frankovich, M.P.Ashworth & M.J.Sullivan |  | Majewska4C |  |
| *Medlinella amphoroidea* Frankovich, M.P.Ashworth & M.J.Sullivan |  | Majewska18C |  |
| *Meuniera membranacea* (Cleve) P. C. Silva | HK313 | ECT3896 (Port Aransas Jetty, Texas) | KC309482, KC309554, KC309624 |
| *Navicula cari* Ehrenberg |  |  | AM501991, AM710457, N/A |
| *Navicula cryptocephala* Kützing | HK090 | FD109 (UTEX) | HQ912603, HQ912467, HQ912296 |
| *Navicula hippodontofallax* Witkowski & Ch. Li |  | SZCZCH703 | KT943636, KT943661, KT943695 |
| *Navicula perminuta* Østrup | HK561 | West Indian Manatee (Crystal River, Florida) | N/A, MT432484, MT432502 |
| *Navicula perminuta* Østrup |  | mbccc3 | JQ045340, JQ432375, N/A |
| *Navicula* sp*.* Bory |  | West Indian Manatee FLManM99WGA11 (Florida) |  |
| *Navicula* sp*.* Bory |  | West Indian Manatee FLMan1cfTur2 (Florida) |  |
| *Navicula* sp*.* Bory | HK486 | Coz4 (Cozumel, Mexico) | MH040322, MH040271, MH040244 |
| *Navicula* sp*.* Bory | HK487 | Coz4 (Cozumel, Mexico) | N/A, MH064103, MH064010 |
| *Navicula* sp*.* Bory | HK488 | 24IV14-2A (Conch Reef, Florida) | MH063475, MH064104, MH064011 |
| *Navicula* sp*.* Bory | HK489 | 24IV14-3A (Pickles Reef, Florida) | MH063476, MH064105, MH064012 |
| *Navicula* sp*.* Bory | HK490 | GU7Y-4 (University of Guam Marine Laboratories, Guam) | N/A, MH040272, MH040245 |
| *Navicula* sp*.* Bory | HK491 | 17VIII13-2 (Belfast, Maine) | MT441512, MT432483, MT432503 |
| *Navicula* sp*.* Bory | HK500 | West Indian Manatee (Georgia) | MH017641, MN977810, MN977815 |
| *Navicula* sp*.* Bory | HK558 | West Indian Manatee (Crystal River, Florida) | N/A, MN977809, MN977814 |
| *Navicula* sp*.* Bory | HK559 | West Indian Manatee (Crystal River, Florida) | MN977831, MN977808, MN977813 |
| *Navicula* sp*.* Bory | HK623 | Loggerhead turtle (Florida Bay, Florida) |  |
| *Navicula* sp*.* Bory | HK624 | Loggerhead turtle (Florida Bay, Florida) |  |
| *Navicula* sp*.* Bory | HK625 | Green sea turtle (Turtle Hospital, Marathon, Florida) |  |
| *Navicula* sp*.* Bory | HK626 | Loggerhead turtle (Florida Bay, Florida) |  |
| *Navicula* sp*.* Bory | HK627 | Diamondback terrapin (Cocoa Beach, Florida) |  |
| *Navicula* sp*.* Bory | HK628 | Diamondback terrapin (Cocoa Beach, Florida) |  |
| *Navicula* sp*.* Bory | HK629 | Diamondback terrapin (Cocoa Beach, Florida) |  |
| *Navicula* sp*.* Bory | KSA0102 | SA4 (Durrah, Saudi Arabia) | KX981844, KX981821, KX981797 |
| *Navicula* sp*.* Bory | KSA0112 | SA23 (Al-Wajh, Saudi Arabia) | N/A, MH064106, MH064013 |
| *Navicula* sp*.* Bory |  | Majewska29A |  |
| *Navicula* sp*.* Bory |  | PMFTB0035 |  |
| *Navicula* sp*.* Bory | UTKSA0131 | KSA2015-19 (Al-Nawras, Jeddah, Saudi Arabia) | MH063477, MH064107, MH064014 |
| *Navicula* sp*.* Bory | UTKSA0162 | KSA2015-14 (Bhadur Resort, Saudi Arabia) | MH063478, MH064108, MH064015 |
| *Navicula* sp*.* Bory | UTKSA0211 | KSA2015-54 (Duba, Saudi Arabia) | MH063469, MH064094, MH064002 |
| *Navicula* sp*.* Bory | UTKSA0239 | KSA2015-41 (Rabigh, Saudi Arabia) | MH063479, MH064109, MH064016 |
| *Navicula reinhardtii* Grunow in Cleve & Möller |  |  | AM501976, AM710442, N/A |
| *Navicula tripunctata* (O.F. Müller) Bory |  |  | AM502028, AM710495, N/A |
| *Navicula zhengii* Witkowski & Li |  | SZCZCH96 | KT943632, KT943681, KT943691 |
| *Neidium affine* (Ehrenberg) Pfitzer | HK064 | FD127 (UTEX) | HQ912583, HQ912447, HQ912276 |
| *Neidium bisulcatum* (Lagerstedt) Cleve | HK076 | FD417 (UTEX) | HQ912591, HQ912455, HQ912284 |
| *Neidium productum* (W. Smith) Cleve | HK063 | FD116 (UTEX) | HQ912582, HQ912446, HQ912275 |
| *Nitzschia acidoclinata* Lange-Bertalot |  |  | KC736632, KC736602, N/A |
| *Nitzschia aurariae* Cholnoky |  | SZCZCH966 | KT943639, KT943663, KT943698 |
| *Nitzschia celaenoae* Lobban, Ashworth, Calaor & Theriot | KSA0035 | SA4 (Durrah, Saudi Arabia) | KU179128, KU179116, KU179143 |
| *Nitzschia draveillensis* Coste & Ricard |  |  | KC736635, KC736605, N/A |
| *Nitzschia dubiformis* Hustedt |  |  | AB430616, AB430696, N/A |
| *Nitzschia inconspicua* Grunow |  |  | KC736636, KC736607, N/A |
| *Nitzschia filiformis* (W. Smith) Van Heurck | HK073 | FD267 (UTEX) | HQ912589, HQ912453, HQ912282 |
| *Nitzschia* cf *frigida* Grunow | HK468 | AKIce (Barrow, Alaska) | N/A, MH064110, MH064017 |
| *Nitzschia frustulum* (Kützing) Grunow |  | TCC545 | KT072974, KT072922, N/A |
| *Nitzschia* cf *longissima* (Brébisson in Kützing) Grunow | HK176 | ECT3689 (Sala Glula, Guam) | KX981850, KX981829, KX981804 |
| *Nitzschia longissima* (Brébisson in Kützing) Grunow |  |  | AY881968, AY881967, N/A |
| *Nitzschia longissima* (Brébisson in Kützing) Grunow | UTKSA0021 | SA29 (Jeddah, Saudi Arabia) | MH063480, MH064111, MH064018 |
| *Nitzschia longissima* (Brébisson in Kützing) Grunow | UTKSA0124 | KSA2015-9 (Bhadur Resort, Saudi Arabia) | MH063481, MH064112, MH064019 |
| *Nitzschia lorenziana* Grunow |  |  | KC736637, KC736608, N/A |
| *Nitzschia martiana* (C. Agardh) Van Heurck | HK405 | 3VIII07 (Talofofo Bay, Guam) | N/A, KJ577899, KJ577933 |
| *Nitzschia* sp*.* Hassall | KSA0120 | SA27 (Jeddah, Saudi Arabia) | KX981849, KX981828, KX981803 |
| *Nitzschia* sp*.* Hassall | HK469 | Rincon Mangrove (Costa Rica) | MH040323, MH040273, MH040246 |
| *Nitzschia* sp*.* Hassall | HK470 | Nate Site 1 (Kona, Hawaii) | MH040324, MH040274, MH040247 |
| *Nitzschia* sp*.* Hassall | HK472 | Coz4 (Cozumel, Mexico) | MH040325, N/A, MH040248 |
| *Nitzschia* sp*.* Hassall | HK474 | CCMP1698 (NCMA) | MH040327, MH040276, MH040250 |
| *Nitzschia* sp*.* Hassall | HK562 | West Indian Manatee (Crystal River, Florida) |  |
| *Nitzschia* sp*.* Hassall |  | SZCZCH658 |  |
| *Nitzschia* sp*.* Hassall | UTKSA0053 | SA19 (Al-Wajh, Saudi Arabia) | N/A, MH064113, MH064020 |
| *Nitzschia* sp*.* Hassall | UTKSA0102 | KSA2015-14 (Bhadur Resort, Saudi Arabia) | MH063482, MH064114, MH064021 |
| *Nitzschia* sp*.* Hassall | UTKSA0106 | KSA2015-49 (Duba, Saudi Arabia) | MH063483, MH064115, MH064022 |
| *Nitzschia* sp*.* Hassall | UTKSA0107 | KSA2015-49 (Duba, Saudi Arabia) | MH063484, MH064116, MH064023 |
| *Nitzschia* sp*.* Hassall | UTKSA0109 | KSA2015-16 (Al-Nawras, Jeddah, Saudi Arabia) | MH063485, MH064117, N/A |
| *Nitzschia* sp*.* Hassall | UTKSA0111 | KSA2015-23 (Markaz Al Shoaibah, Saudi Arabia) | MH063486, MH064118, MH064024 |
| *Nitzschia* sp*.* Hassall | UTKSA0133 |  |  |
| *Nitzschia* sp*.* Hassall | UTKSA0143 |  |  |
| *Nitzschia* sp*.* Hassall | UTKSA0171 | KSA2015-11 (Bhadur Resort, Saudi Arabia) | MH063487, MH064119, MH064025 |
| *Nitzschia* sp*.* Hassall | UTKSA0173 | KSA2015-37 (Rabigh, Saudi Arabia) | MH063488, MH064120, MH064026 |
| *Nitzschia* sp*.* Hassall | UTKSA0182 | KSA2015-38 (Rabigh, Saudi Arabia) | MH063489, MH064121, MH064027 |
| *Nitzschia* sp*.* Hassall | UTKSA0260 | KSA2015-11 (Bhadur Resort, Saudi Arabia) | MH063490, MH064122, MH064028 |
| *Nitzschia traheaformis* Ch. Li, Witkowski & Yu Sh. |  | SZCZCH970 | KT943642, KT943666, KT943701 |
| *Nitzschia traheaformis* Ch. Li, Witkowski & Yu Sh. |  | SZCZCH971 | KT943643, KT943667, KT943702 |
| *Nitzschia volvendirostrata* Ashworth, Dabek & Witkowski | KSA0039 | SA12 (Markaz Al Shoaibah, Saudi Arabia) | N/A, KU179112, KU179139 |
| *Parlibellus hamulifer* (Grunow) Cox | HK409 | GU44AK-4 (Gab Gab Beach, Guam) | KJ577866, KJ577903, KJ577937 |
| *Parlibellus* cf *hamulifer* (Grunow) Cox | HK428 | SantaRosaCor.green (Costa Rica) | KU179137, KU179122, KU179149 |
| *Parlibellus harffianus* Witkowski, Ch. Li & S.-X.Yu |  | SZCZCH75 | KT943652, KT943686, KT943715 |
| *Parlibellus* sp. E.J. Cox | UTKSA0240 |  |  |
| *Phaeodactylum tricornutum* Bohlin | HK011 | CCMP2561 (NCMA) | HQ912556, HQ912420, HQ912250 |
| *Phaeodactylum tricornutum* Bohlin | HK538 | UTEX640 (UTEX) | MH063492, MH064125, MH064031 |
| *Phaeodactylum tricornutum* Bohlin | HK539 | UTEX646 (UTEX) | MH063493, MH064126, MH064032 |
| *Phaeodactylum tricornutum* Bohlin | HK540 | UTEX2089 (UTEX) | MH063494, MH064127, MH064033 |
| *Pinnularia brebissonii* (Kützing) Rabenhorst | HK092 | FD274 (UTEX) | HQ912604, HQ912468, HQ912297 |
| *Pinnularia termitina* (Ehrenberg) Patrick | HK088 | FD484 (UTEX) | HQ912601, HQ912465, HQ912294 |
| *Poulinea lepidochelicola* Majewska, De Stefano & Van de Vijver | HK554 | Green Sea turtle (Turtle Hospital, Marathon, Florida) |  |
| *Poulinea lepidochelicola* Majewska, De Stefano & Van de Vijver | HK555 | Green Sea turtle (Turtle Hospital, Marathon, Florida) |  |
| *Poulinea lepidochelicola* Majewska, De Stefano & Van de Vijver | HK556 | Green Sea turtle (Turtle Hospital, Marathon, Florida) |  |
| *Poulinea lepidochelicola* Majewska, De Stefano & Van de Vijver | HK630 | RM July19 Wasabi (Durban, South Africa) |  |
| *Poulinea lepidochelicola* Majewska, De Stefano & Van de Vijver | HK631 | Olive ridley turtle (Turtle Hospital, Marathon, Florida) |  |
| *Poulinea lepidochelicola* Majewska, De Stefano & Van de Vijver | HK638 | RM July19 Wasabi (Durban, South Africa) |  |
| *Poulinea lepidochelicola* Majewska, De Stefano & Van de Vijver | HK639 | RM July19 Shiv (Durban, South Africa) |  |
| *Poulinea lepidochelicola* Majewska, De Stefano & Van de Vijver | HK640 | RM July19 Shiv (Durban, South Africa) |  |
| *Poulinea lepidochelicola* Majewska, De Stefano & Van de Vijver | HK676 | Olive ridley turtle (Aquarium of the Pacific, California) |  |
| *Poulinea lepidochelicola* Majewska, De Stefano & Van de Vijver |  | PMFTB0073 |  |
| *Poulinea lepidochelicola* Majewska, De Stefano & Van de Vijver |  | PMFTB0074 |  |
| *Poulinea lepidochelicola* Majewska, De Stefano & Van de Vijver |  | PMFTB0077 |  |
| *Poulinea lepidochelicola* Majewska, De Stefano & Van de Vijver |  | Majewska5A |  |
| *Poulinea lepidochelicola* Majewska, De Stefano & Van de Vijver |  | Majewska14C |  |
| *Poulinea lepidochelicola* Majewska, De Stefano & Van de Vijver |  | Majewska17C |  |
| *Poulinea lepidochelicola* Majewska, De Stefano & Van de Vijver |  | Majewska20C |  |
| *Placoneis elginensis* (Gregory) Cox | HK096 | FD416 (UTEX) | HQ912607, HQ912471, HQ912300 |
| *Plagiotropis* sp*.* Pfitzer | HK508 | PR5 (Condado Lagoon, Puerto Rico) | MH063495, MH064128, MH064034 |
| *Planothidium frequentissimum* (Lange-Bertalot) Lange-Bertalot |  | PF1 | KJ658409, KJ658392, N/A |
| *Planothidium lanceolatum* (Brébisson ex Kützing) Lange-Bertalot |  | PL2 | KJ658410, KJ658393, N/A |
| *Planothidium* sp*.* Round & Bukhtiyarova |  | SZCZCH26 | KT943653, KT943678, KT943716 |
| *Pleurosigma* sp*.* W. Smith | HK495 | GU52X-1 (Outhouse Beach, Guam) | MH040327, MH040276, MH040250 |
| *Pleurosigma* sp*.* W. Smith | UTKSA0019 | SA18 (Duba, Saudi Arabia) | KX981840, KX981822, KX981798 |
| *Pleurosigma* sp*.* W. Smith | UTKSA0167 | KSA2015-49 (Duba, Saudi Arabia) | MH063496, MH064129, MH064035 |
| *Pleurosigma* sp*.* W. Smith | UTKSA0264 | KSA2015-16 (Al-Nawras, Jeddah, Saudi Arabia) | MH063497, N/A, MH064036 |
| *Pleurosigma* sp*.* W. Smith | UTKSA0273 | KSA2015-16 (Al-Nawras, Jeddah, Saudi Arabia) | MH063498, MH064130, MH064037 |
| *Pleurosigma stuxbergii* Cleve & Grunow |  | SZCZCH973 | N/A, KT943674, KT943711 |
| *Proschkinia* cf *complanatula* (Hustedt ex Simonsen) D.G. Mann | HK553 | 24II18-1G (Half Moon Bay, California) | MK736943, MK757575, MK757579 |
| *Proschkinia impar* So-Yeon Kim, J.-G. Park & Witkowski |  | KNU-Y-16121 | N/A, MK887893, N/A |
| *Proschkinia luticola* So-Yeon Kim, J.-G. Park, Witkowski & B.-S. Kim |  | KNU-B-16024 | N/A, MK887894, N/A |
| *Proschkinia modesta* So-Yeon Kim, J.-G. Park & Witkowski |  | KNU-Y-16122 | N/A, MK887895, N/A |
| *Proschkinia staurospeciosa* So-Yeon Kim, J.-G. Park, Witkowski & Gastineau |  | SZCZR1824 | N/A, MK887897, N/A |
| *Proschkinia sulcata* Majewska, Van de Vijver & Bosak |  | PMFTB0019 |  |
| *Proschkinia vergostriata* Frankovich, Ashworth & M.J. Sullivan | HK548 | Loggerhead turtle (Florida Bay, Florida) | N/A, MK757570, N/A |
| *Proschkinia vergostriata* Frankovich, Ashworth & M.J. Sullivan | HK549 | Loggerhead turtle (Florida Bay, Florida) | MK736939, MK757571, N/A |
| *Proschkinia vergostriata* Frankovich, Ashworth & M.J. Sullivan | HK550 | Green Sea turtle (Turtle Hospital, Marathon, Florida) | MK736940, MK757572, MK757576 |
| *Proschkinia vergostriata* Frankovich, Ashworth & M.J. Sullivan | HK551 | Green Sea turtle (Turtle Hospital, Marathon, Florida) | MK736941, MK757573, MK757577 |
| *Proschkinia vergostriata* Frankovich, Ashworth & M.J. Sullivan | HK552 | Green Sea turtle (Turtle Hospital, Marathon, Florida) | MK736942, MK757574, MK757578 |
| *Proschkinia vergostriata* Frankovich, Ashworth & M.J. Sullivan | HK642 | Green Sea turtle (Bahamas) |  |
| *Psammodictyon constrictum* (Gregory) Mann in Round, Crawford & Mann | HK440 | GU7X-7 (University of Guam Marine Lab, Guam) | KX981851, KX981830, KX981805 |
| *Psammodictyon constrictum* (Gregory) Mann in Round, Crawford & Mann | HK471 | Nate Site 1 (Kona, Hawaii) | MH040329, MH040278, MH040252 |
| *Psammodictyon* sp*.* D.G. Mann | UTKSA0117 | KSA2015-30 (Markaz Al Shoaibah, Saudi Arabia) | MH063499, MH064131, MH064038 |
| *Psammodictyon* sp*.* D.G. Mann | UTKSA0151 | KSA2015-37 (Rabigh, Saudi Arabia) | MH063500, MH064132, MH064039 |
| *Psammodictyon* sp*.* D.G. Mann | UTKSA0280 | KSA2015-2 (Bhadur Resort, Saudi Arabia) | MH063501, MH064133, MH064040 |
| *Psammodictyon pustulatum* (Voigt ex Meister) Lobban | UTKSA0298 | KSA2015-38 (Rabigh, Saudi Arabia) | MH063502, MH064134, MH064041 |
| *Pseudogomphonema* sp. Medlin | HK560 | 25II18-1B (Garrapata State Park, California) |  |
| *Rhoiconeis pagoensis* C.S. Lobban | HK419 | GU7X-7 (University of Guam Marine Lab, Guam) | KX981846, KX981825, KX981800 |
| *Rhoiconeis pagoensis* C.S. Lobban | UTKSA0128 | KSA2015-16 (Al-Nawras, Jeddah, Saudi Arabia) | MH063503, MH064135, N/A |
| *Rhoicosigma* sp*.* Grunow | UTKSA0194 | KSA2015-22 (Markaz Al Shoaibah, Saudi Arabia) | MH063504, MH064136, MH064042 |
| *Rhoicosphenia abbreviata* (C.Agardh) Lange-Bertalot |  | CH030 | KJ011672, KJ011854, N/A |
| *Rhoicosphenia* cf *abbreviata* (C.Agardh) Lange-Bertalot |  | EWT2016.80 | KU965569, KU965580, N/A |
| *Rhopalodia contorta* Hustedt |  | L1299 (UTEX) | HQ912406, HQ912392, HQ912378 |
| *Rhopalodia gibba* (Ehrenberg) O. Müller |  |  | HQ912407, HQ912393, HQ912379 |
| *Rhopalodia* sp*.* O. Müller | HK433 | 21IV14-4D (Rabbit Key Basin, Florida) | KX981843, KX981823, KX981799 |
| *Rhopalodia* sp*.* O. Müller |  | ECT3678 (Tinago River, Guam) | HQ912405, HQ912391, HQ912377 |
| *Rossia* sp*.* Voigt |  |  | EF151968, EF143281, N/A |
| *Schizostauron rawaii* Ashworth, J. Sabir & Witkowski | UTKSA0141 | KSA2015-11 (Bhadur Resort, Saudi Arabia) | MH063505, MH064137, MH064043 |
| *Schizostauron kajotkei* Dąbek, Górecka & Witkowski |  | SZCZP32 | KT943595, KT943606, KT943619 |
| *Schizostauron kajotkei* Dąbek, Górecka & Witkowski |  | SZCZP40 | KT943596, KT943607, KT943620 |
| *Scoliopleura peisonis* Grunow | HK103 | FD13 (UTEX) | HQ912609, HQ912473, HQ912302 |
| *Sellaphora laevissima* (Kützing) D.G.Mann |  | THR4 | EF151981, EF143309, N/A |
| *Sellaphora minima* Grunow |  | TCC524 | KF959656, KF959642, N/A |
| *Sellaphora seminulum* (Grunow) D.G. Mann |  | TCC461 | KF959642, KC736613, N/A |
| *Seminavis robusta* D.B.Danielidis & D.G.Mann | HK492 | GU7X-7 (University of Guam Marine Laboratories, Guam) | MH040330, MH040279, MH040253 |
| *Stauroneis acuta* W. Smith | HK059 | FD51 (UTEX) | HQ912579, HQ912443, HQ912272 |
| *Stauroneis anceps* Ehrenberg |  |  | AM502008, AM710475, N/A |
| *Stauroneis gracilior* Reichardt |  |  | AM501988, AM710454, N/A |
| *Stauroneis kriegeri* Patrick |  |  | AM501990, AM710456, N/A |
| *Stauroneis phoenicentron* (Nitzsch) Ehrenberg |  |  | AM502031, AM710498, N/A |
| *Staurophora* sp. Mereschkowsky | HK503 |  |  |
| *Staurotropis americana* Ashworth | HK442 | FishPassMangrove (Mustang Island, Texas) | KX981855, KX981834, KX981808 |
| *Staurotropis americana* Ashworth | HK443 | Coz4 (Cozumel, Mexico) | KX981854, KX981833, KX981807 |
| *Staurotropis khiyamii* J. Sabir & Ashworth | UTKSA0047 | SA18 (Duba, Saudi Arabia) | KX981853, KX981832, KX981806 |
| *Staurotropis seychellensis* (Giffen) Paddock | HK172 | ECT3721 (University of Guam Marine Lab, Guam) | KX981856, N/A, KX981809 |
| *Stenopterobia curvula* (W. Smith) Krammer |  | L541 (UTEX) | HQ912416, HQ912402, HQ912388 |
| *Sternimirus shandongensis* Witkowski & Li |  | SZCZCH968 | KT943637, KT943662, KT943696 |
| *Surirella* cf *fastuosa* (Ehrenberg) Ehrenberg |  | SZCZCH189 | KT943629, KT943655, KT943688 |
| *Surirella minuta* Van Heurck |  | FD320 (UTEX) | HQ912658, HQ912522, HQ912351 |
| *Surirella ovata* Kützing | HK214 | L1241 (UTEX) | HQ912658, HQ912522, HQ912351 |
| *Surirella splendida* (Ehrenberg) Kützing |  |  | HQ912415, HQ912401, HQ912387 |
| *Surirella* sp*.* Turpin | UTKSA0299 | KSA2015-2 (Bhadur Resort, Saudi Arabia) | MH063507, MH064139, MH064045 |
| *Tetramphora chilensis* (Hustedt) Stepanek & Kociolek |  | AMPH132 | KU665638, KU665639, KU665640 |
| *Trachyneis* sp*.* P.T. Cleve | HK439 | SantaRosaCor.green (Costa Rica) | KX981845, KX981824, N/A |
| *Tryblionella apiculata* Gregory | HK087 | FD465 (UTEX) | HQ912600, HQ912464, HQ912293 |
| *Tryblionella gaoana* Witkowski & Ch. Li |  | SZCZCH97 | KT943638, KT943683, KT943697 |
| *Tryblionella* sp. W. Smith | HK501 | West Indian Manatee (Crystal River, Florida) |  |
| *Tursiocola* *alata* Frankovich, Ashworth & M.J. Sullivan |  | West Indian Manatee FLManM99WGA7 (Florida) | MH017646, N/A, N/A |
| *Tursiocola* *alata* Frankovich, Ashworth & M.J. Sullivan |  | West Indian Manatee FLManM99WGA8 (Florida) | MH017647, N/A, N/A |
| *Tursiocola* *alata* Frankovich, Ashworth & M.J. Sullivan |  | West Indian Manatee FLManM99WGA21 (Florida) | MH017648, N/A, N/A |
| *Tursiocola* *bondei* Frankovich, Ashworth & M.J. Sullivan |  | West Indian Manatee FLManM117TurWGA9 (Florida) | MH017649, N/A, N/A |
| *Tursiocola* *bondei* Frankovich, Ashworth & M.J. Sullivan |  | West Indian Manatee FLManM117TurWGA10 (Florida) | MH017650, N/A, N/A |
| *Tursiocola denysii* Frankovich & M.J. Sullivan | HK633 | Green sea turtle (Bahamas) |  |
| *Tursiocola denysii* Frankovich & M.J. Sullivan | HK643 | Green sea turtle (Bahamas) |  |
| *Tursiocola guyanensis* Riaux-Gobin & A. Witkowski | HK632 | Green sea turtle (Florida) |  |
| *Tursiocola* sp. R.W.Holmes, S.Nagasawa & H.Takano |  | West Indian Manatee FLMan50cfTur24 (Florida) | MH017651, N/A, N/A |
| *Tursiocola* *varicopulifera* Frankovich & M.J. Sullivan |  | West Indian Manatee FLManM95WGA1 (Florida) | MH017653, N/A, N/A |
| *Tursiocola* *varicopulifera* Frankovich & M.J. Sullivan |  | West Indian Manatee FLManM95WGA2 (Florida) | MH017654, N/A, N/A |
| *Tursiocola* *varicopulifera* Frankovich & M.J. Sullivan |  | West Indian Manatee FLManM99WGA22 (Florida) | MH017655, N/A, N/A |
| *Tursiocola* *varicopulifera* Frankovich & M.J. Sullivan |  | West Indian Manatee FLManM99WGA23 (Florida) | MH017656, N/A, N/A |
| *Tursiocola* cf *ziemanii* Frankovich & M.J. Sullivan |  | West Indian Manatee CGA1605TTcostWGA4 (Georgia) |  |
| *Tursiocola* cf *ziemanii* Frankovich & M.J. Sullivan |  | West Indian Manatee CGA1605TTcostWGA5 (Georgia) | MH017665, N/A, N/A |
| *Tursiocola* cf *ziemanii* Frankovich & M.J. Sullivan |  | West Indian Manatee CGA1605TTcostWGA8 (Georgia) | MH017666, N/A, N/A |
| *Tursiocola* cf *ziemanii* Frankovich & M.J. Sullivan |  | West Indian Manatee FLMan42cfTur11 (Florida) | MH017661, N/A, N/A |
| *Tursiocola* cf *ziemanii* Frankovich & M.J. Sullivan |  | West Indian Manatee FLMan42cfTur18 (Florida) | MH017662, N/A, N/A |
| *Tursiocola* cf *ziemanii* Frankovich & M.J. Sullivan |  | West Indian Manatee FLMan42cfTur19 (Florida) | MH017663, N/A, N/A |
| *Tursiocola* cf *ziemanii* Frankovich & M.J. Sullivan |  | West Indian Manatee FLMan49cfTur22 (Florida) | MH017664, N/A, N/A |
| *Tursiocola* *ziemanii* Frankovich & M.J. Sullivan |  | West Indian Manatee FLManM99WGA4 (Florida) | MH017657, N/A, N/A |
| *Tursiocola* *ziemanii* Frankovich & M.J. Sullivan |  | West Indian Manatee FLManM99WGA10 (Florida) | MH017658, N/A, N/A |
| *Tursiocola* *ziemanii* Frankovich & M.J. Sullivan |  | West Indian Manatee FLManM99WGA12 (Florida) | MH017659, N/A, N/A |
| unidentified monoraphid | UTKSA0152 | KSA2015-37 (Rabigh, Saudi Arabia) | MH063509, MH064141, MH064047 |
| unidentified monoraphid | UTKSA0158 | KSA2015-37 (Rabigh, Saudi Arabia) | MH063510, MH064142, N/A |
| unidentified naviculoid | HK497 | 23X15-5B (Harbor Branch Oceanographic Institute boat launch) | MH063511, MH064143, MH064048 |
| unidentified naviculoid | UTKSA0247 | KSA2015-5 (Bhadur Resort, Saudi Arabia) | MH063512, MH064144, MH064049 |
| unidentified stauroneid | UTKSA0220 | KSA2015-7 (Bhadur Resort, Saudi Arabia) | MH063513, MH064145, MH064050 |
| Araphid Outgroups |  |  |  |
| *Asterionella formosa* Hassall | HK144 | UTCC605 | HQ912633, HQ912497, HQ912326 |
| *Asterionellopsis glacialis* (Castracane) Round | HK107 | CCMP134 (NCMA) | HQ912613, HQ912477, HQ912306 |
| *Asterionellopsis socialis* (Lewin & Norris) Crawford & Gardner | HK181 | CCMP1717 (NCMA) | HQ912646, HQ912510, HQ912339 |
| *Asterionellopsis socialis* (Lewin & Norris) Crawford & Gardner | HK319 | ECT3920 (Ft. Stevens State Park, Oregon) | JX413545, JX413562, JX413579 |
| *Astrosyne radiata* Ashworth & Lobban | HK169 | ECT3697 (Gab Gab Beach, Guam) | JN975238, JN975252, JN975267 |
| *Bleakeleya notata* (Grunow in Van Heurck) F.E. Round | HK247 | ECT3733 (Pago Bay, Guam) | HM627330, HM627327, HM627324 |
| *Castoridens hyalina* Ashworth, Witkowski & Li | HK444 | C1 12-7-13 (Destin-Choctawhatchee Bay, Florida) | N/A, KU851892, KU851907 |
| *Castoridens striata* Ashworth, Li & Witkowski | HK385 | 15VI11-2A (Baffin Bay, Texas) | KJ577844, KJ577879, KJ577915 |
| *Catacombas gaillonii* (Bory de Saint-Vincent) Williams & Round |  | s0045 | KR048195, KR048217, KR048229 |
| *Centronella reicheltii* Voigt | HK150 | CCAP1011/1 | HQ912635, HQ912499, HQ912328 |
| *Ctenophora pulchella* (Ralfs ex Kützing) Williams & Round | HK105 | FD150 (UTEX) | HQ912611, HQ912475, HQ912304 |
| *Cyclophora castracanei* Ashworth & Lobban | HK243 | GU44AB-6 (Gab Gab Beach, Guam) | JN975242, JN975256, JN975271 |
| *Cyclophora castracanei* Ashworth & Lobban | HK395 | GU44AN-7 (Gab Gab Beach, Guam) | KJ577854, KJ577889, N/A |
| *Cyclophora* cf *minor* Ashworth & Lobban | HK461 | 24IV14-3A (Pickles Reef, Florida) | MH040308, MH040254, MH040230 |
| *Cyclophora tabellariformis* Ashworth & Lobban | HK306 | ECT3892 (Carrabelle, Florida) | JN975243, JN975257, JN975272 |
| *Cyclophora tabellariformis* Ashworth & Lobban | HK460 | GU44AY-6 (Gab Gab Beach, Guam) | MH040309, MH040255, N/A |
| *Cyclophora tenuis* Castracane | HK216 | ECT3723 (Umatac Bay, Guam) | HQ912660, HQ912524, HQ912353 |
| *Cyclophora tenuis* Castracane | HK307 | ECT3854 (Kahana Beach Park, Oahu, Hawaii) | JN975240, JN975254, JN975269 |
| *Cyclophora tenuis* Castracane | HK308 | ECT3838 (Long Beach, California) | JN975241, JN975255, JN975270 |
| *Delphineis surirella* (Ehrenberg) G.W. Andrews | HK133 | CCMP1095 | HQ912629, HQ912493, HQ912322 |
| *Delphineis surirella* (Ehrenberg) G.W. Andrews | HK295 | ECT3886 (Bald Head Island, North Carolina) | JX413544, JX413561, JX413578 |
| *Diatoma elongata* (Lyngbye) C.Agardh | HK119 | UTCC62 | HQ912622, HQ912486, HQ912315 |
| *Diatoma tenue* Agardh | HK078 | FD106 (UTEX) | HQ912593, HQ912457, HQ912286 |
| *Dimeregramma* sp*.* J. Ralfs in A. Pritchard | HK288 | ECT3864 (MSI, Port Aransas, Texas) | JN975244, JN975258, JN975273 |
| *Dimeregramma* sp*.* J. Ralfs in A. Pritchard | HK358 | 15VI11-2A (Baffin Bay, Texas) | JX401231, JX401249, JX401267 |
| *Dimeregramma* sp*.* J. Ralfs in A. Pritchard | HK359 | ECT3891 (St. George Island, Florida) | JX401232, JX401250, JX401268 |
| *Dimeregramma* sp*.* J. Ralfs in A. Pritchard | HK376 | 25VI12-1C (Hunting Island, South Carolina) | KF701596, KF701605, KF701614 |
| *Dimeregramma* sp*.* J. Ralfs in A. Pritchard | HK377 | AtlanticPlankton#8 (Florida) | KF701597, KF701606, KF701615 |
| *Florella pascuensis* Navarro | HK175 | ECT3756 (Guam) | JN975246, JN975260, JN975275 |
| *Fragilariforma virescens* (Ralfs) Williams & Round | HK132 | FD291 (UTEX) | HQ912628, HQ912492, HQ912321 |
| *Grammatophora macilenta* W. Smith | HK368 | GU44AK-4 (Gab Gab Beach, Guam) | JX401241, JX401259, JX401276 |
| *Grammatophora oceanica* Ehrenberg | HK147 | CCMP410 | HQ912634, HQ912498, HQ912327 |
| *Grammatophora* sp*.* Ehrenberg | HK459 | Nate Site 1 (Hawaii) | MG684352, MG684323, MG684295 |
| *Grammatophora* sp*.* Ehrenberg | UTKSA0132 | KSA2015-16 (Al-Nawras, Jeddah, Saudi Arabia) | MH063514, MH064146, MH064051 |
| *Grammatophora undulata* Ehrenberg | HK367 | Coz-3 (Cozumel, Mexico) | JX401240, JX401258, JX401275 |
| *Grammonema striatula* (Lyngbye) Agardh | HK371 | ECT3897 (Pebble Beach, California) | KF701591, KF701600, KF701609 |
| *Hanicella moenia* Lobban & Ashworth | HK379 | GU44AK-6 (Gab Gab Beach, Guam) | KF701599, KF701608, KF701617 |
| *Hendeyella dimeregrammopsis* Ashworth | HK391 | Coz-1 (Cozumel, Mexico) | KJ577850, KJ577885, KJ577920 |
| *Hendeyella lineata* Ashworth & Lobban | HK325 | GU44AI-3 (Gab Gab Beach, Guam) | JX413547, JX413564, JX413581 |
| *Koernerella recticostata* (Körner) Ashworth, Lobban & Theriot | HK242 | GU44AB-8 (Gab Gab Beach, Guam) | HM627331, HM627328, HM627325 |
| *Licmophora abbreviata* Agardh | UTKSA0049 | SA29 (Jeddah, Saudi Arabia) | KP125882, KP125883, KP125884 |
| *Licmophora colosalis* Belando, Aboal & Jiménez | HK366 | ECT3907 (Rabbit Key Basin, Florida) | JX401239, JX401257, JX401274 |
| *Licmophora colosalis* Belando, Aboal & Jiménez | UTKSA0066 | SA29 (Jeddah, Saudi Arabia) | MG684358, MG684329, MG684299 |
| *Licmophora* aff *ehrenbergii* (Kützing) Grunow | HK420 | GU7X-6 (University of Guam Marine Lab, Guam) | KP125876, KP125879, KP125881 |
| *Licmophora flucticulata* Lobban, Schefter & Ruck |  | GU56-A (Cocos Wall, Guam) | HQ997923, JN975262, JN975277 |
| *Licmophora normaniana* (Greville) Wahrer in Wahrer, Fryxell & Cox | HK403 | 26II12-1 (Mustang Island, Texas) | KJ577860, KJ577897. KJ577931 |
| *Licmophora paradoxa* (Lyngbye) Agardh | HK106 | CCMP2313 | HQ912612, HQ912476, HQ912305 |
| *Licmophora peragallioides* (Lobban) Lobban & Ashworth | HK364 | GU44AL-3 (Gab Gab Beach, Guam) | JX401237, JX401255, JX401273 |
| *Licmophora* sp*.* Agardh | HK302 | GU52-O (Outhouse Beach, Guam) | JN975248, JN975263, N/A |
| *Licmophora* sp*.* Agardh | HK365 | Coz-2 (Cozumel, Mexico) | JX401238, JX401256, N/A |
| *Licmophora* sp*.* Agardh | KSA0085 | SA4 (Durrah, Saudi Arabia) | MG684353, MG684324, N/A |
| *Licmophora* sp. Agardh | KSA0151 | SA1 (Durrah, Saudi Arabia) | MG684354, MG684325, N/A |
| *Licmophora* sp. Agardh | UTKSA0010 | SA29 (Jeddah, Saudi Arabia) | MG684355, MG684326, MG684296 |
| *Licmophora* sp. Agardh | UTKSA0029 | SA18 (Duba, Saudi Arabia) | MG684356, MG684327, MG684297 |
| *Licmophora* sp. Agardh | UTKSA0050 | SA18 (Duba, Saudi Arabia) | MG684357, MG684328, MG684298 |
| *Licmophora* sp. Agardh | UTKSA0084 | SA18 (Duba, Saudi Arabia) | MG684359, MG684330, MG684300 |
| *Licmophora* sp. Agardh | UTKSA0191 | KSA2015-14 (Bhadur Resort, Saudi Arabia) | MH063515, MH064147, MH064052 |
| *Lucanicum concatenatum* Lobban & Ashworth | HK378 | GU44AI-3 (Gab Gab Beach, Guam) | KF701598, KF701607, KF701616 |
| *Microtabella interrupta* (Ehrenberg) Round | HK248 | ECT3700 (Gab Gab Beach, Guam) | JN975247, JN975261, JN975276 |
| *Microtabella interrupta* (Ehrenberg) Round | HK458 | 20X15-1 (Boca Chica Channel, Florida) | MH040319, MH040265, MH040238 |
| *Nanofrustulum* cf *shiloi* (J.J. Lee, C.W. Reimer, & M.E. McEnery) F.E. Round, H. Hallsteinsen, & E. Paasche | HK056 | CCMP2649 | HQ912578, HQ912442, HQ912271 |
| *Neodelphineis* sp*.* Takano | HK421 | FijiBottleNY (New York) | KP125875, KP125878, N/A |
| *Neofragilaria nicobarica* Desikachary, Prasad & Prema |  | s0371 | AB433340, KR048216 KR048228 |
| *Neofragilaria* cf *nicobarica* Desikachary, Prasad & Prema | HK375 | Coz-1 (Cozumel, Mexico) | KF701595, KF701604, KF701613 |
| *Neosynedra provincialis* (Grunow) Williams & Round | HK457 | 24IV14-3A (Pickles Reef, Florida) | N/A, MH040266, MH040239 |
| *Opephora guenter-grassi* (Witkowski & Lange-Bertalot) Sabbe & Vyverman |  | s0263 | AB436781, KR048218, N/A |
| *Opephora pacifica* (Grunow) Petit | HK296 | ECT3831 (Ward Island, Texas) | JN975249, JN975264, JN975278 |
| *Perideraion elongatum* Jordan, Arai & Lobban | HK411 | GU44AK-6 (Gab Gab Beach, Guam) | KJ577868, KJ577905, KJ577939 |
| *Perideraion* cf *elongatum* Jordan, Arai & Lobban | UTKSA0259 | KSA2015-49 (Duba, Saudi Arabia) | MH063516, MH064148, MH064053 |
| *Perideraion montgomeryii* Lobban, Jordan & Ashworth | HK246 | GU7 (University of Guam Marine Lab, Guam) | HM627332, HM627329, HM627326 |
| *Plagiogramma porcipellis* Ashworth & Chunlian Li | HK212 | ECT3776 (Taeleyag Beach, Guam) | HQ912656, HQ912520, HQ912349 |
| *Plagiogramma* sp*.* Greville | HK324 | ECT3924 (Potlatch State Park, Washington) | JX413546, JX413563, JX413580 |
| *Plagiogramma* sp*.* Greville | HK374 | 25VI12-1C (Hunting Island, South Carolina) | KF701594, KF701603, KF701612 |
| *Plagiostriata goreensis* Sato & Medlin |  | s0388 | KR048198, KR048220, KR048232 |
| *Psammogramma vigoensis* Sato & Medlin |  | s0391 | KR048194, KR048215, KR048227 |
| *Psammoneis japonica* Sato, Kooistra & Medlin | HK299 | GU52-O (Outhouse Beach, Guam) | JN975250, JN975265, JN975279 |
| *Psammoneis obaidii* Ashworth & Sabir | UTKSA0057 | SA12 (Markaz Al Shoaibah, Saudi Arabia) | KR059023, KR059022, KR059024 |
| *Psammoneis* sp*.* Sato, Kooistra & Medlin | UTKSA0250 | KSA2015-42 (Rabigh, Saudi Arabia) | MH063517, MH064149, N/A |
| *Psammotaenia lanceolata* Ashworth, Li & Witkowski | HK316 | 10X10-2 (St. George Island, Florida) | JX413543, JX413560, JX413577 |
| *Pseudostriatella oceanica* Sato, Mann & Medlin |  | s0384 | KR048197, KR048219, KR048231 |
| *Pteroncola* sp*.* R.W. Holmes & D.A. Croll | UTKSA0078 | SA29 (Jeddah, Saudi Arabia) | MG684376, N/A, MG684316 |
| *Podocystis* cf *americana* Bailey | HK453 | 19X15-1A (Channel #5, Florida) | MH040320, MH040267, MH040240 |
| *Podocystis* cf *americana* Bailey | HK454 | 19X15-1B (Channel #5, Florida) | MG684360, MG684331, MG684301 |
| *Podocystis spathulata* (Shadbolt) Van Heurck | HK217 | ECT3733 (Pago Bay, Guam) | HQ912661, HQ912525, HQ912354 |
| *Rhabdonema adriaticum* Kützing | HK370 | Coz-3 (Cozumel, Mexico) | JX401243, JX401261, JX401278 |
| *Rhabdonema arcuatum* (Lyngbye) Kützing | HK304 | ECT3898 (Pebble Beach, California) | JN975251, JN975266, JN975280 |
| *Rhabdonema* sp*.* Kützing | HK369 | GU44AI-1 (Gab Gab Beach, Guam) | JX401242, JX401260, JX401277 |
| *Rhaphoneis amphiceros* (Ehrenberg) Ehrenberg | HK237 | ECT3828 (Redfish Bay, Texas) | HQ912673, HQ912537, KC309625 |
| *Rhaphoneis amphiceros* (Ehrenberg) Ehrenberg | HK373 | 25VI12-1A (Hunting Island, South Carolina) | KF701593, KF701602, KF701611 |
| *Serratifera* sp*.* Li, Ashworth & Witkowski |  | Majweska2C  Hawksbill turtle (Durban, South Africa) |  |
| *Serratifera varisterna* Li, Ashworth & Witkowski | HK315 | 9X10-2 (Florida State University Marine Lab, Florida) | JX413542, JX413559, JX413576 |
| *Serratifera varisterna* Li, Ashworth & Witkowski | HK424 | PackaryChannelPlankton (Mustang Island, Texas) | KU851868, KU851879, KU851894 |
| *Staurosira construens* Ehrenberg | HK071 | FD232 (UTEX) | HQ912587, HQ912451, HQ912280 |
| *Staurosirella pinnata* (Ehrenberg) Williams & Round | HK116 | CCMP330 (NCMA) | HQ912620, HQ912484, HQ912313 |
| *Striatella unipunctata* (Lyngbye) Agardh | HK177 | ECT3648 (Asan Beach, Guam) | HQ912643, HQ912507, HQ912336 |
| *Striatella unipunctata* (Lyngbye) Agardh | HK318 | ECT3874 (Channel #5, Florida) | JX419383, JX419384, JX419385 |
| *Stricosus blumbergii* Theriot & Ashworth | HK362 | 15VI11-2A (Baffin Bay, Texas) | JX401235, JX401253, JX401271 |
| *Stricosus harrisonii* Lobban & Theriot | HK363 | GU44AI (Gab Gab Beach, Guam) | JX401236, JX401254, JX401272 |
| *Synedra famelica* Kützing | HK072 | FD255 (UTEX) | HQ912588, HQ912452, HQ912281 |
| *Synedra ulna* (Nitzsch) Ehrenberg | HK075 | FD404 (UTEX) | HQ912590, HQ912454, HQ912283 |
| *Synedropsis hyperborea* (Grunow) Hasle, Medlin & Syvertsen | HK117 | CCMP1423 (NCMA) | HQ912621, HQ912485,  HQ912314 |
| *Synedropsis* cf *recta* Hasle, Medlin & Syvertsen | HK110 | CCMP1620 (NCMA) | HQ912616, HQ912480, HQ912309 |
| *Tabellaria flocculosa* (Roth) Kützing | HK065 | FD133 (UTEX) | HQ912584, HQ912448, HQ912277 |
| *Tabularia* cf *tabulata* (Agardh) Snoeijs | HK109 | CCMP846 (NCMA) | HQ912615, HQ912479, HQ912308 |
| *Talaroneis posidoniae* Kooistra & De Stefano | WK59 |  | AY216905, KR048214, KR048226 |
| *Tetracyclus* sp. Ralfs | HK416 | B12 (Lake Baikal, Russia) | KJ577873, KJ577910, KJ577944 |
| *Thalassionema* cf *bacillare* (Heiden) Kolbe | HK361 | ECT3929 (Gulf of Mexico, Texas) | JX401234, JX401252, JX401270 |
| *Thalassionema frauenfeldii* (Grunow) Tempère & Peragallo | HK372 | 25VI12-1A (Hunting Island, South Carolina) | KF701592, KF701601, KF701610 |
| *Thalassionema* cf *nitzschioides* (Grunow) Mereschkowsky | HK360 | ECT3929 (Gulf of Mexico, Texas) | JX401233, JX401251, JX401269 |
