# Supplementary material for: Cultivating epizoic diatoms provides insights into the evolution and ecology of both epibionts and hosts
Source: Sci Rep. 2022 Sep 6;12:15116. doi: 10.1038/s41598-022-19064-0 (PMC9448772; doi:10.1038/s41598-022-19064-0)
Supplement: Supplementary file 4 — Supplementary Legends. [file 41598_2022_19064_MOESM4_ESM.docx]

**Supplementary Information**

Supplemental Figure S1. Complete Maximum Likelihood phylogenetic tree derived from a concatenated 3-gene DNA sequence dataset. Support values (ML bootstrap support) shown above nodes. Taxa isolated from epizoic habitats followed by a diagrammatic representation of the host from which the strain was isolated, and metadata on the location and setting in which the host was sampled (A = aquarium, R = rehabilitation facility, W = wild). Black host icon = POE taxon; white host icon = unclear habitat preference.

Supplemental Figure S2. Complete Bayesian Inference phylogenetic tree derived from a concatenated 3-gene DNA sequence dataset. Support values (BI posterior probability) shown above nodes. Taxa isolated from epizoic habitats followed by a diagrammatic representation of the host from which the strain was isolated, and metadata on the location and setting in which the host was sampled (A = aquarium, R = rehabilitation facility, W = wild). Black host icon = POE taxon; white host icon = unclear habitat preference.

Supplemental Table S1. Taxa, strain voucher ID and GenBank accession numbers for strains used in the DNA sequence data phylogenetic analysis. Collection site for sample of original strain isolation, or culture collection strain number, is also included (where known). Ingroup taxa (raphid pennates) provided first in the table; outgroup taxa (araphid pennates) follow after table break. Taxa are listed alphabetically. If species unknown, authority for genus is listed.
